# Utilization of delactosed whey permeate for the synthesis of ethyl acetate with *Kluyveromyces marxianus*

**DOI:** 10.1007/s00253-023-12419-1

**Published:** 2023-02-14

**Authors:** Andreas Hoffmann, Alexander Franz, Thomas Walther, Christian Löser

**Affiliations:** 1 Bioprocess Engineering, Institute of Natural Materials Technology, Technische Universität Dresden, 01062 Dresden, Germany; 2 Biophysical Chemistry, Institute of Biochemistry, University of Leipzig, 04103 Leipzig, Germany

**Keywords:** Delactosed whey permeate, Iron limitation, Fed-batch cultivation, Repeated-batch cultivation, Ethyl acetate

## Abstract

**Abstract:**

Ethyl acetate is an important organic solvent and currently produced from fossil carbon resources. Microbial synthesis of this ester from sugar-rich waste could be an interesting alternative. Therefore, synthesis of ethyl acetate by *Kluyveromyces marxinanus* DSM 5422 from delactosed whey permeate (DWP) was studied in an aerated stirred bioreactor at 40 °C. DWP is mainly composed of residual lactose and minerals. The minerals inhibited yeast growth, as witnessed by an increased lag period, a reduced growth rate, and an extended process duration. All experiments were therefore carried out with diluted DWP. In a series of batch experiments, the pH of iron-deficient DWP medium varied between 4.8 and 5.9. The pH of the cultivation medium significantly influenced cell growth and product syntheses, with the highest ethyl acetate yield of 0.347 g g^–1^ and lowest by-product formation achieved at pH 5.1. It is likely that this effect is due to pH-dependent iron chelation, which affects the iron bioavailability and the intracellular iron content, thus affecting growth and metabolite synthesis. The viability of yeast cells was always high despite the harsh conditions in DWP medium, which enabled extended usage of the biomass in repeated-batch and fed-batch cultivations. These two culture techniques increased the volume of DWP processed per time by 32 and 84% for the repeated-batch and the fed-batch cultivation, respectively, without a drop of the ester yield.

**Key points:**

*• Delactosed whey permeate was converted to ethyl acetate with a high rate and yield.*

*• The formation of ethyl acetate in DWP medium at iron limitation is pH-dependent.*

*• Highly active yeasts from batch processes enabled extension as fed and repeated batch.*

**Graphical Abstract:**

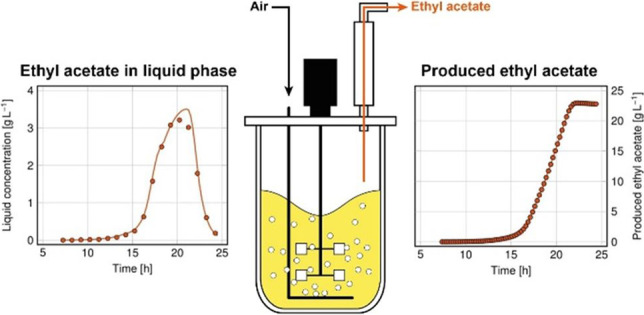

**Supplementary Information:**

The online version contains supplementary material available at 10.1007/s00253-023-12419-1.

## Introduction

Ethyl acetate is a commodity chemical with versatile industrial applications: as dissolver in chemical reactions, for extractive and chromatographic product recovery, cleaning surfaces, processing surface formulations, and for the production of adhesives, print colors, and paints (Löser et al. 2014; Posada et al. 2013). Ethyl acetate is currently produced from fossil resources such as natural gas by energy-intensive petrochemical processes (Löser et al. 2014; Nielsen et al. 2012). However, the more sustainable microbial synthesis of ethyl acetate from sugar-rich waste streams shows high economic potential (Straathof and Bampouli 2017). Production of ethyl acetate from concentrated whey permeates (CWPs) by *Kluyveromyces marxianus* has been successfully demonstrated (Löser et al. 2013; Urit et al. 2011, 2012, 2013a, b). These CWPs arise during whey processing: sweet and sour whey is microfiltrated for extracting whey proteins, the obtained permeate is concentrated by reverse osmosis, and partially demineralized, if applicable by precipitation of calcium phosphate via slight alkalization and warming. CWP is, however, a valuable resource for lactose production: CWP is concentrated once more by evaporation of water under vacuum followed by lactose crystallization via cooling, creating a delactosed whey permeate (DWP) that still contains 150 to 200 g L^‒1^ residual lactose (Friend et al. 2004; Wagner et al. 2014) and 90% of the whey-borne minerals (Durham and Hourigan 2007). DWP is in part used as animal feed and fertilizer but often regarded as waste because of the high mineral load (Durham 2009).

Several biotechnological approaches have been considered for valorization of DWP, such as production of ethanol (Shen et al. 2019; Wagner et al. 2014), acetoin (Liu et al. 2020), succinic acid (Banger et al. 2021), and lactic acid (AgriChemWhey 2022), but fermentation of DWP to ethanol is currently the only industrial application (Cloidt et al. 2008).

Biotechnological utilization of DWP is complicated by compositional variability (Oliveira et al. 2019; Online Resource 1) and the high mineral content, especially K^+^, Na^+^, Ca^2+^, and Cl^‒^ ions (Banger et al. 2021; Friend et al. 2004; Liang et al. 2009), which inhibits microbial growth (Maddox 1995; Mahmoud and Kosikowski 1982). Reducing the mineral content of DWPs by electrodialysis is not practicable due to the high costs of this technology. Another way to alleviate growth inhibition by minerals is dilution with water prior to fermentation, but this reduces the achievable product concentration (Wagner et al. 2014) and increases the costs for downstream processing (Galbe et al. 2007).

Production of ethyl acetate from DWP could be an interesting alternative. Ethyl acetate is highly volatile and therefore quickly transferred from the culture broth to the gas phase, thus leaving the bioreactor with the exhaust gas (Löser et al. 2021; Urit et al. 2011). This stripping counteracts accumulation of the ester in the liquid phase and avoids product inhibition (Urit et al. 2013b). Moreover, synthesis of the ester and its extraction from the culture broth occurs simultaneously. The separation of ethyl acetate from the exhaust gas may be performed by adsorption; ethyl acetate is easily adsorbed by activated carbon and zeolites (Manjare and Ghoshal 2006a, b, c) and is thus separable from exhaust gas streams (Medeiros et al. 2006). Alternatively, ethyl acetate is separable by membrane technology (Hoffmann et al. 2022). Combination of microbial synthesis of ethyl acetate with innovative downstream processing to recover the product offers thus an option for the microbial valorization of DWP.

The best-known wild-type producers of ethyl acetate are the yeasts *Cyberlindnera jadinii*, *Wickerhamomyces anomalus*, and *K. marxianus*, in particular the strain *K. marxianus* DSM 5422 (Hoffmann et al. 2021; Kruis et al. 2018; Löser et al. 2011; Online Resource 2). Synthesis of ethyl acetate is induced by suboptimal growth conditions such as iron limitation (Armstrong and Yamazaki 1984; Armstrong et al. 1984; Hoffmann et al. 2021; Kallel-Mhiri et al. 1993; Kruis et al. 2018; Löbs et al. 2017; Löser et al. 2011, 2012, 2013; Urit et al. 2011, 2012, 2013a, b; Willetts 1989), copper limitation (Urit et al. 2012), and oxygen limitation (Armstrong et al. 1984; Fredlund et al. 2004; Hoffmann et al. 2021; Kallel-Mhiri et al. 1993; Kruis et al. 2017; Löser et al. 2015a; Rojas et al. 2001).

Limitation by iron or copper reduces the activity of the electron transport chain (ETC) since all ETC complexes depend on iron and complex IV depends on copper. A limited availability of oxygen as the terminal electron acceptor diminishes the ETC activity as well. The decreased ETC activity slows down the mitochondrial re-oxidation of NADH, resulting in a lack of NAD^+^ and a diminished flux of acetyl-CoA into the Krebs cycle (Kruis et al. 2017; Löbs et al. 2018; Löser et al. 2012, 2015a). Accumulation of acetyl-CoA leads to an increased pyruvate level in the cytosol. Pyruvate is partially converted to ethanol, which diffuses into the mitochondria, where it is condensed with acetyl-CoA by the ethanol acetyltransferase 1 (Eat1), yielding ethyl acetate, thus, counteracting the accumulation of mitochondrial acetyl-CoA (Kruis et al. 2018; Löbs et al. 2018; Löser et al. 2015a; Thomas and Dawson 1978).

This mechanism has been confirmed by two observations: the ethyl acetate-forming Eat1 enzyme is localized in the mitochondria of various yeasts (Kruis et al. 2018; Löbs et al. 2018), and exposure of *K. marxianus* to the ETC inhibitors antimycin A, carboxin, and cyanide, acting on complex II, III, or IV, respectively, has triggered synthesis of ethyl acetate in *K. marxianus* (Löser et al. 2015a).

From a practical point of view, synthesis of ethyl acetate is best controlled by reducing iron availability (Hoffmann et al. 2021). Copper limitation is difficult to impose since many feedstocks often contain surplus copper, and the intensity of oxygen limitation is difficult to control, often leading to unwanted ethanol production (Hoffmann et al. 2021; Löser et al. 2015a).

Recent identification of Eat1 as the key enzyme for bulk synthesis of ethyl acetate in yeasts has boosted the development of genetically engineered microbes for an optimized production of ethyl acetate (Bohnenkamp et al. 2020, 2021; Kruis et al. 2017, 2019, 2020; Löbs et al. 2018). Such rational metabolic engineering helps to develop improved producer strains. However, microbial conversion of sugar-rich wastes from the food industry into valuable products is preferably performed by wild-type microbes with GRAS status, based on a lacking customer acceptance for genetic engineering in connection with food. Therefore, microbial production by wild-type strains remains significant also in the future.

In this study, we demonstrate the efficient production of ethyl acetate from delactosed whey permeate (DWP) by the wild-type strain *K. marxianus* DSM 5422 under aerobic and iron-limited conditions. A suitable dilution of DWP is identified for diminishing the inhibitory effect of DWP-borne minerals to a tolerable level. Then, an appropriate pH value is explored since preliminary investigations referred to a pH dependency of the ester synthesis in DWP-based media. Moreover, it is shown that fed-batch and repeated-batch cultivations under the optimized culture conditions can further improve the performance of the process.

## Materials and methods

### Microorganism and culture media

The yeast *Kluyveromyces marxianus* DSM 5422 originates from the Deutsche Sammlung von Mikroorganismen und Zellkulturen GmbH (Germany) and was stored in 50% (*v*/*v*) glycerol at –80 °C. Inocula were obtained by two-day cultivation at 30 °C on YGC agar (Roth GmbH, Germany), containing 20 g L^‒1^ glucose, 5 g L^‒1^ yeast extract, 0.2 g L^‒1^ chloroamphenicol and 15 g L^‒1^ agar.

Delactosed whey permeate (DWP) was provided by the Sachsenmilch Leppersdorf GmbH (Germany). A typical lot of the DWP exhibited the following composition (given in masses per 1 L): 146 g lactose, 19 g citrate, 5*.*3 g proteins, 5.0 g lactate, 4.6 g galactose, 2*.*9 g pyruvate, 0.9 g glycerol, < 1 g succinate, malate, and fumarate, minerals such as 16*.*6 g K^+^, 6*.*7 g Na^+^, 0*.*7 g Mg^2+^, 0*.*6 g Ca^2+^, 13*.*2 g Cl^–^, 6*.*1 g PO_4_^3–^, and 5*.*7 g SO_4_^2–^, and trace elements such as 1*.*2 mg zinc, 0*.*4 mg iron, and 0*.*1 mg copper. Sum parameters were dry matter (266*.*6 g L^‒1^), minerals (49.6 g L^*–*1^), and ash (47*.*5 g L^*–*1^). Protein was measured according to Bradford (1976), small organic molecules were analyzed by HPLC as described below, cations and anions were measured by ion chromatography and inductively coupled plasma mass spectrometry (UFZ Leipzig, Germany), and ash was determined according to Mumm (1970).

The inhibition of growth by whey-borne minerals was studied in DWP-based media. DWP was variably diluted with deionized water and heat-sterilized (121 °C, 20 min). Each 1 L of these dilutions was supplemented with 7 g urea and 2 mL trace-element solution with iron (Urit et al. 2011) to avoid limitation of growth by nitrogen or micronutrients. Both supplements were separately heat sterilized, with urea sterilized in the absence of water to prevent hydrolysis (Löser et al. 2015b). The pH of the media was adjusted to 5.1 by adding 1 M H_2_SO_4_. This acidification somewhat diluted the media but also added sulfate ions, slightly modifying the mineral content of the prepared media.

For all other cultivation experiments, the medium was prepared as follows: 0*.*5 L DWP was diluted with 0*.*5 L deionized water and heat sterilized (121 °C, 20 min). Then, 7 g urea and 2 mL trace-element solution with iron or without iron (Urit et al. 2011) were added and referred to as DWP^+Fe^ or DWP^–Fe^ medium, respectively. The lacking iron acts as an inductor for ester synthesis. These media exhibited a pH of 6.4.

### Bioreactor cultivation

The cultivations were performed in a 3.6-L stirred bioreactor (Labfors 5, Infors GmbH, Switzerland). The reactor was autoclaved for 20 min at 121 °C and then filled with 1 L pre-sterilized DWP-based medium and 0*.*5 mL sterile antifoam (J 673 A, Schill + Seilacher Struktol GmbH, Germany). The bioreactor was operated at 1500 rpm and 40 °C, which is the optimal temperature for this strain (Urit et al. 2013a). The aeration rate was 60 L h^*–*1^ (DWP^–Fe^ medium) or 180 L h^*–*1^ (inhibition experiments), given for 0 °C and 101,325 Pa. The supplied air holds 0*.*008 L L^*‒*1^ water and 0.0004 L L^*–*1^ CO_2_. The pH was controlled to a desired value by supplying 1 M H_2_SO_4_ and 2 M NaOH. The exhaust gas passed a condenser for partial dehumidification (dew point 11*.*5 °C) and was then analyzed regarding O_2_ and CO_2_ by an EL3020 analyzer (ABB, Germany) and regarding volatiles as described below. For inoculation, three loops of biomass from an agar-plate culture were suspended in 2 mL of sterile water, and 1 mL of this suspension was injected into the bioreactor.

Batch cultivations with a pH shift were performed with DWP^–Fe^ medium and operated in two stages. In the first cultivation phase, the pH was controlled to 5*.*1. The second cultivation phase was initiated when the CO_2_ in the exhaust gas exceeded a content of 0.001 L L^*–*1^; then, the set point of the pH controller was switched to another value ranging from pH 4.8 to 5.9.

Fed-batch cultivations started as batch processes based on 1 L DWP^–Fe^ medium, as just described. The feed consisted of another 1 L DWP^–Fe^ medium, which was supplied with a constant rate. Feeding began at a high ester-synthesis rate, and the feeding rate was chosen in such a manner that the lactose concentration remained nearly constant: ${F}_{\text{Feed}}=\mu \cdot {C}_{\text{X}}\cdot {V}_{\text{L}}/({Y}_{{\text{X}}\text{/}{\text{S}}}\cdot ({C}_{\text{S,0}}-{C}_{\text{S}}))$; all variables apply to the moment of starting the fed-batch process (regarding the used symbols, it is referred to Table 1).

**Table 1:** Nomenclature

Symbol	Unit	Description
${C}_{\mathrm{I}}$	g L^-1^	Concentration of an inhibitory compound, here whey-borne minerals
${C}_{\mathrm{I},\mathrm{max}}$	g L^-1^	Parameter of the model of Luong (1985)
${C}_{i,\mathrm{G}}$	g L^-1^	Concentration of compound $i$ in the gas at the gas-line exit
${C}_{i,\mathrm{L}}$	g L^-1^	Concentration of compound $i$ in the cultivation medium
${C}_{\mathrm{S}}$	g L^-1^	Sugar concentration in the cultivation medium
${C}_{\mathrm{S},0}$	g L^-1^	Sugar concentration in the supplied medium during fed-batch cultivation
${C}_{\mathrm{X}}$	g L^-1^	Biomass concentration in the cultivation medium given as dry weight
$\mathrm{CTR}$	mol L^-1^ h^-1^	Carbon dioxide transfer rate
${F}_{\mathrm{Feed}}$	L h^-1^	Flow of medium supplied during fed-batch cultivation
${K}_{i,\mathrm{L}/\mathrm{G}}$	L L^-1^	Liquid–gas partition coefficient of compound $i$ in the bioreactor
${m}_{i}$	*g*	Mass of cumulatively synthesized/degraded compound $i$
$n$	‒	Parameter of the model of Luong (1985)
$\mathrm{OTR}$	mol L^-1^ h^-1^	Oxygen transfer rate
${R}_{\mathrm{i}}$	g L^-1^ h^-1^	Volume-specific rate of synthesized/degraded compound $i$
$\mathrm{RQ}$	mol mol^-1^	Respiratory quotient
${r}_{i}$	g g^-1^ h^-1^	Biomass-specific rate of synthesized/degraded compound $i$
${V}_{L}$	L	Volume of the cultivation medium in the bioreactor
${x}_{\mathrm{Fe}}$	g g^-1^	Iron content of the biomass
${Y}_{i/\mathrm{S}}$	g g^-1^	Yield of compound $i$ related to the substrate
${Y}_{\mathrm{EA}/\mathrm{S},\mathrm{max}}$	g g^-1^	Maximum yield of ethyl acetate
${Y}_{\mathrm{X}/\mathrm{S}}$	g g^-1^	Yield of yeast growth
$\mu$	h^-1^	Specific growth rate
$\psi$	g g^-1^ h^-1^	Biomass-specific iron-uptake rate
Indices ($i$)		Description
$\mathrm{AA}$		Compound acetaldehyde
$\mathrm{Ac}$		Compound acetate
$\mathrm{EA}$		Compound ethyl acetate
$\mathrm{EtOH}$		Compound ethanol
$\mathrm{VOC}$		Volatile organic compounds

Repeated-batch cultivations also started as a batch process using 1 L DWP^–Fe^ medium as described above. After depletion of the sugar (becoming visible from a steep decline in CO_2_ formation), 0.9 L cell suspension was replaced by 0.9 L fresh DWP^–Fe^ medium using a peristaltic pump. Then the second batch phase was conducted under the same conditions as before.

### Analytical methods

The biomass dry weight was determined by separating yeast from the suspension via centrifugation, washing the pellet twice with deionized water, and drying at 105 °C until weight constancy.

The proportion of dead cells in yeast populations was determined by propidium iodide (PI) staining followed by fluorescence measurement using a Cyflow Cube 8 flow cytometer (Partec GmbH, Germany) equipped with a 20-mW 488-nm solid-state laser. Samples were diluted with PBS buffer (8 g NaCl, 0.2 g KCl, 1.42 g Na_2_HPO_4_, and 0.27 g KH_2_PO_4_ in 1 L water, yielding pH 7.4) to OD_600nm_ ≈ 0.05. Then, 975 µL diluted cell suspension was mixed with 25 µL 80 µM PI, incubated for 15 min at 25 °C in the dark, and the fluorescence was analyzed at 565 to 615 nm.

Small organic molecules were quantified by a UltiMate3000 HPLC (Thermo Fisher Scientific, USA) equipped with a Rezex ROA-Organic acid H + column (Phenomenex LTD, Germany). The column was operated at 65 °C with 0*.*3 mL min^*–*1^ 2.5 mM H_2_SO_4_ as an eluent. Lactose, galactose, and glycerol were measured with a RefractoMax 520 detector (ERC, Germany), and acids were quantified with a DAD-3000(RS) detector at 210 nm (Thermo Fisher Scientific, USA). Samples were prepared prior to injection as follows: 150 µL sample were mixed with 150 µL methanol, stored at – 20 °C for 20 min to precipitate proteins, and centrifuged in a MiniSpin Plus system (Eppendorf, Germany) at 14,500 rpm for 10 min. Two hundred microliters of the supernatant was diluted with 800 µL deionized water and filtered (0*.*45 µm, regenerated cellulose). Lastly, an amount of 20 µL filtrate was injected.

The content of volatile organic compounds (VOCs) in the liquid and gas phase (${C}_{\text{VOC,L}}$ and ${C}_{\text{VOC,G}}$) were analyzed by gas chromatography according to (Urit et al. 2011). Gas phase concentrations were also quantified by a GAM 2000 mass spectrometer (InProcess Instruments, Germany). The volume-specific and biomass-specific reaction rates (${R}_{\mathrm{VOC}}$ and ${r}_{\mathrm{VOC}}$) and the masses of formed VOCs (${m}_{\text{VOC}}$) were calculated as described in (Löser et al. 2021) with two modifications: (a) the impact of stripped VOCs on the flow of the exhaust gas was taken into account as demonstrated by (Urit et al. 2013b) for ethyl acetate; (b) the partition coefficients of VOCs (*K*_VOC*,*L*/*G_) were calculated from measured *C*_VOC*,*L_ and ${C}_{\text{VOC,G}}$ concentrations after conversion of the *C*_VOC*,*G_ data into headspace concentrations (Löser et al. 2021), done individually for each experiment. This was necessary due to differences in the used DWP batches. The following average values were obtained: ${K}_{\mathrm{EA},\mathrm{L}/\mathrm{G}}$ = 53 ± 6 L L^–1^, ${K}_{\mathrm{AA},\mathrm{L}/\mathrm{G}}$ = 144 ± 23 L L^–1^, and ${K}_{\mathrm{EtOH},\mathrm{L}/\mathrm{G}}$ = 1657 ± 115 L L^–1^. The masses of stripped compounds were calculated from the ${C}_{\text{VOC,G}}(t)$ data until ${C}_{\text{EA,G}}$ fell below 1 mg L^‒1^ (typically 1 h after sugar depletion).

The impact of dosage of pH correctives, medium feed, evaporation of water, and sampling during the process on the liquid volume and liquid-phase concentrations was taken into account by calculation as described in Löser et al. (2018). All confidence intervals of data were calculated for a confidence level of 95%.

The selectivity of ester synthesis was calculated from the mass of synthesized ethyl acetate divided by the sum of the masses of all formed metabolites. Similarly, the selectivity of ester stripping was calculated from the mass of stripped ethyl acetate divided by the sum of the masses of all stripped metabolites.

## Results

### Inhibition of growth by whey-borne minerals

Delactosed whey permeate (DWP) has a high content of whey-borne sugars, organic acids, and minerals. Dissolved minerals significantly inhibit yeast growth (Maddox 1995; Mahmoud and Kosikowski 1982). On the other hand, growth inhibition depends on several factors, such as specific medium composition, culture conditions (temperature and pH), and cultivated species and strain. The inhibitory effect of a particular whey-borne medium has thus to be examined individually for a selected yeast strain and the chosen culture conditions.

Here, the growth-inhibiting effect of a DWP on *K. marxianus* DSM 5422 was investigated under aerobic conditions at pH 5.1 and 40 °C. The mineral content was used as benchmark criterion for the growth rate because it was expected to have the greatest impact of all DWP components on growth. The mineral concentration was varied by using different DWP/water ratios, with a volume fraction of 10, 50, 75, or 100% DWP, resulting in a mineral content of 5.8, 26.4, 39.0, or 51.6 g L^–1^. The cultivations were performed in a bioreactor to avoid limitation by oxygen. The specific growth rates were determined from exhaust gas data as described by Löser et al. (2021), which is demonstrated for one process in Online Resource 3.

**Fig. 1:**
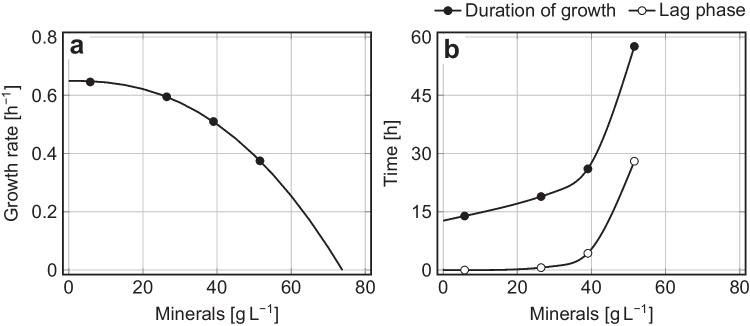
Inhibition of yeast growth by whey-borne minerals during aerobic batch cultivation of *K. marxianus* DSM 5422 in stirred bioreactors using 1 L differently diluted DWP; DWP was diluted with water and supplemented with 7 g L^‒1^ urea and trace-element solution with iron; cultivation at 40 °C, aeration with 180 L h^‒1^, and pH 5.1; **a **Specific growth rate depending on the mineral content of pre-diluted DWP media fitted with the inhibition model of Luong (1985): $\mu =\mu ({C}_{I}\text{=}0)\cdot (1-{({C}_{I}/{C}_{I,max})}^{n})$ with $\mu ({C}_{I}\text{=}0)$ = 0*.*65 h^*–*1^, ${C}_{I,max}$ = 73.8 g L^‒1^, and $n$ = 2.4 as model parameters; **b **Duration of the cultivation process until lactose depletion and duration of the lag phase

An increasing mineral content gradually diminished the growth rate of the yeast (Fig. 1a). Nevertheless, *K. marxianus* DSM 5422 even grew in non-diluted DWP with a rate of 0.375 h^‒1^, which was 58% of the growth rate at negligible inhibition in medium with 10% DWP. Furthermore, a high mineral content also caused an extended lag phase which contributed to longer overall process durations (Fig. 1b).

A long cultivation process complicates experimentation in the laboratory (unfeasible prolongation of the observation and sampling period) and—much more importantly—is economically disadvantageous. Therefore, DWP was systematically diluted 1:1 (*v/v*) with water to a total salt concentration of 26.4 g L^‒1^ in all experiments described below.

### Influence of the pH on iron-limited batch cultivations

The effect of pH on the ester synthesis of *K. marxianus* DSM 5422 was studied in a stirred bioreactor using DWP^–Fe^ medium. This medium is based on DWP, which was diluted 1:1 (v/v) with water and supplemented with urea and trace elements without iron. Aeration with air at 1 vvm ensured a dissolved oxygen content of > 40% oxygen saturation throughout the process. Each cultivation started at pH 5.1 and was automatically changed to the desired pH value when the CO_2_ content of the exhaust gas exceeded 0.001 L L^‒1^ (corresponding to $\mathrm{CTR}$ = 1.6 mmol L^-1^ h^-1^). The initial cultivation at pH 5.1 allowed reproducible growth within the first hours of all processes, which helped to better schedule the sampling period for all individual processes, since the pH affects yeast growth, as demonstrated exemplarily for pH 5.9 (Online Resource 4).

**Fig. 2:**
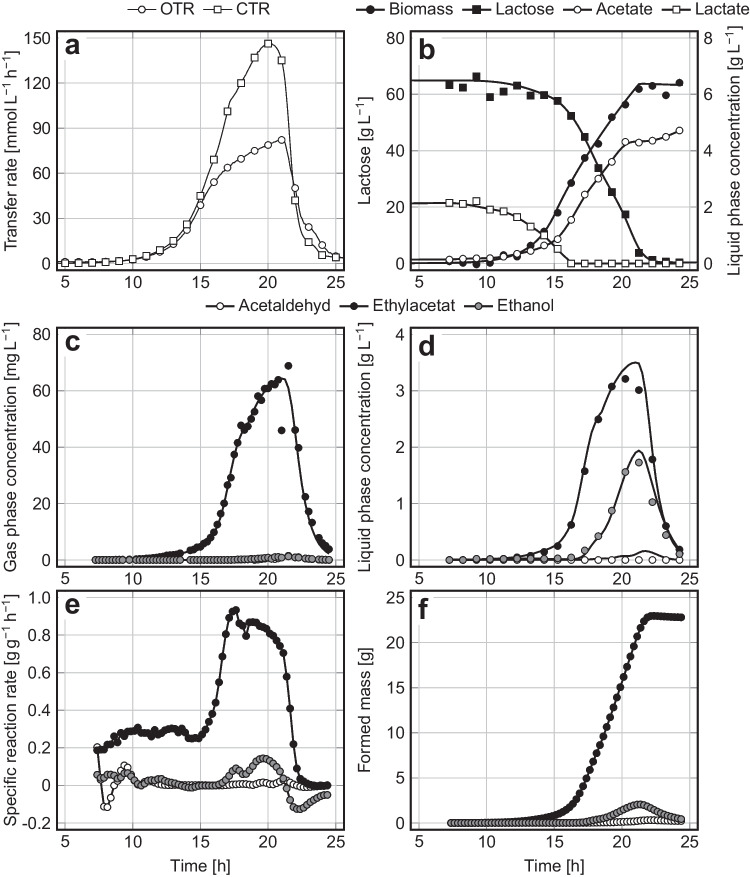
**a **Oxygen transfer rate ($OTR$) and CO_2_ transfer rate ($CTR$); **b **lactose, biomass, acetate, and lactate concentration; **c **gas phase concentrations, **d **liquid phase concentrations, **e **biomass-specific reaction rates, and **f **masses of formed ethyl acetate, ethanol, and acetaldehyde during the aerobic batch cultivation of *K. marxianus* DSM 5422 under iron-limited conditions in a stirred bioreactor using 1 L DWP^‒Fe^ medium; cultivation at 40 °C, aeration with 60 L h^‒1^, and pH 5.1

Five pH values ranging from 4.8 to 5.9 were tested, and the results are shown in Table 2. It becomes clearly visible that all calculated parameters of cell growth and product syntheses were more or less influenced by the pH. The best results were achieved at pH 5.1, at which most ethyl acetate was produced at the highest synthesis rate, combined with low biomass and by-product formation and a low process duration. In detail, lactate and lactose were co-metabolized within the first 16.2 h of cultivation, and only little ethyl acetate was formed. After lactate depletion, the synthesis of ethyl acetate steadily intensified, and formation of acetate increased. Synthesis of some ethanol started after 16.4 h, while production of acetaldehyde was negligible. After 22.8 h, the sugar was depleted (Fig. 2). Due to its permanent stripping from the aerated bioreactor, the concentrations of ethyl acetate in the culture medium did not exceed 4 g L^‒1^. In contrast, ethanol was hardly stripped but remained in the culture medium and was consumed during a later stage of cultivation. Regarding the different stripping behavior of ethyl acetate and ethanol, it is referred to Löser et al. (2021). All further experiments were conducted at pH 5.1.

**Table 2:** Parameters of cell growth and product synthesis during aerobic cultivations of *K. marxianus* DSM 5422 in a stirred bioreactor under iron-limited conditions using DWP^‒Fe^ or DWP^+Fe^ medium; cultivation at 40 °C, aeration with 60 L h^–1^, and pH values as indicated in the table header (pH shifts were performed at a CO_2_ content of 0.001 L L^–1^ in the exhaust gas); further details of the cultivations are given in the captions of Figs. 2, 3, and 4; n. d. means not determined

Cultivation medium	DWP^+Fe^	DWP^‒Fe^						
Cultivation mode	Batch	Batch with a pH shift from 5.1 to …					Fed batch	Repeated batch
pH	5.1 ^a^	4.8	5.1	5.4	5.65	5.9	5.1	5.1
Process time till depletion of sugars [*h*]	18.9	29.1	22.8	23.1	27.3	25.4	24.8	32.8
Final proportion of living cells [%]	n.d	97.3	97.8	96.4	96.7	98.8	96.6	97.9
Average respiratory quotient, $RQ$ [mol mol^-1^]	1.07	1.28	1.48	1.69	1.81	1.84	1.58	1.70
Final biomass concentration [g L^-1^]	24.3	5.98	6.37	7.87	8.80	10.10	6.80	6.09
Maximum ${C}_{\mathrm{EA},\mathrm{G}}$ [mg L^-1^]	1.5	56.5	64.4	60.4	52.3	52.4	48.8	66.5
Maximum ${C}_{\mathrm{EA},\mathrm{L}}$ [g L^-1^]	0.08	2.26	3.50	3.12	2.22	2.36	2.58	3.18
Mass of formed ethyl acetate, ${m}_{\mathrm{EA}}$ [g]	0.63	15.4	23.0	20.3	17.6	16.5	43.5	45.0
Mass of stripped ethyl acetate[g]	0.60	14.6	22.6	19.9	16.6	15.4	41.6	43.4
Maximum ${R}_{\mathrm{EA}}$ [g L^-1^ h^-1^]	0.33	4.24	4.65	4.38	3.99	4.31	3.47	4.67
Maximum ${r}_{\mathrm{EA}}$ [g g^-1^ h^-1^]	0.05	0.70	0.93	0.95	0.63	0.50	0.70	0.94
Overall yield of ethyl acetate, ${Y}_{\mathrm{EA}/\mathrm{S}}$ [g g^-1^]	0.009	0.245	0.347	0.302	0.327	0.255	0.311	0.368
Y_ES/S_-Y_EA/S,max_ ratio [%]	1.7	47.5	67.4	58.6	63.5	49.5	60.4	71.5
Selectivity of ester formation [g g^-1^]	n.d	0.555	0.765	0.690	0.659	0.554	0.750	0.738
Selectivity of ester stripping [g g^-1^]	n.d	0.863	0.978	0.944	0.867	0.823	0.951	0.951
Maximum ${C}_{\mathrm{EtOH},\mathrm{G}}$ [mg L^-1^]	0.02	1.91	1.20	3.13	3.14	5.00	1.71	3.84
Maximum ${C}_{\mathrm{EtOH},\mathrm{L}}$ [g L^-1^]	< 0.01	3.08	1.94	4.19	4.40	7.26	2.57	5.58
Mass of formed ethanol, ${m}_{\mathrm{EtOH}}$ [g]	0.03	3.53	2.06	4.47	5.12	8.35	5.72	8.13
Mass of stripped ethanol [g]	0.01	1.24	0.29	0.70	1.19	1.67	0.95	1.37
Maximum ${R}_{\mathrm{EtOH}}$ [g L^-1^ h^-1^]	0.04	0.92	0.78	2.14	1.55	4.20	1.10	1.76
Maximum ${r}_{\mathrm{EtOH}}$ [g g^-1^ h^-1^]	0.06	0.15	0.14	0.33	0.78	0.76	0.20	0.35
Overall yield of ethanol, ${Y}_{\mathrm{EtOH}/\mathrm{S}}$ [g g^-1^]	< 0.01	0.06	0.03	0.07	0.09	0.13	0.04	0.07
Maximum ${C}_{\mathrm{AA},\mathrm{G}}$ [mg L^-1^]	< 0.01	3.03	1.15	3.02	4.83	5.88	2.07	4.31
Maximum ${C}_{\mathrm{AA},\mathrm{L}}$ [g L^-1^]	< 0.01	0.42	0.16	0.50	0.53	0.65	0.27	0.48
Mass of formed acetaldehyde, ${m}_{\mathrm{AA}}$ [g]	< 0.01	1.06	0.31	0.78	1.76	2.12	1.56	1.46
Mass of stripped acetaldehyde [g]	< 0.01	1.07	0.23	0.48	1.36	1.59	1.19	0.88
Maximum ${R}_{\mathrm{AA}}$ [g L^-1^ h^-1^]	< 0.01	0.32	0.18	4.38	0.53	1.28	0.22	0.76
Maximum ${r}_{\mathrm{AA}}$ [g g^-1^ h^-1^]	< 0.01	0.05	0.20	0.08	0.19	0.15	0.30	0.13
Overall yield of acetaldehyde, ${Y}_{\mathrm{AA}/\mathrm{S}}$ [g g^-1^]	< 0.01	0.02	< 0.01	0.01	0.03	0.03	0.01	0.01
Maximum ${C}_{\mathrm{Ac},\mathrm{L}}$ [g L^-1^]	0.33	7.82	4.75	3.87	2.30	2.80	3.69	4.19
Mass of formed acetate, ${m}_{\mathrm{Ac}}$ [g]	0.33	7.76	4.67	3.88	2.23	2.79	7.27	6.36
Overall yield of acetate, ${Y}_{\mathrm{Ac}/\mathrm{S}}$ [g g^-1^]	< 0.01	0.12	0.07	0.06	0.04	0.04	0.05	0.05

^a^ Oxygen limitation occurred 1.5 h before the sugar was depleted owing to the high biomass concentration in this experiment. Only the aerobic part of the process was considered for quantifying the process parameters

### Iron-limited fed-batch cultivation

Synthesis of ethyl acetate from DWP^–Fe^ medium is optimal at pH 5.1 (Table 2), but the period of vigorous ester synthesis was relatively short due to depletion of sugar (Fig. 2). The phase of intensive ester production was therefore prolonged by fed-batch cultivation. The feeding phase started after 18.3 h, when the synthesis rate of ethyl acetate was high. A feeding rate of 0.37 L h^‒1^ was chosen to ensure a nearly constant sugar concentration during the feeding period (calculated as described in the methods chapter).

**Fig. 3:**
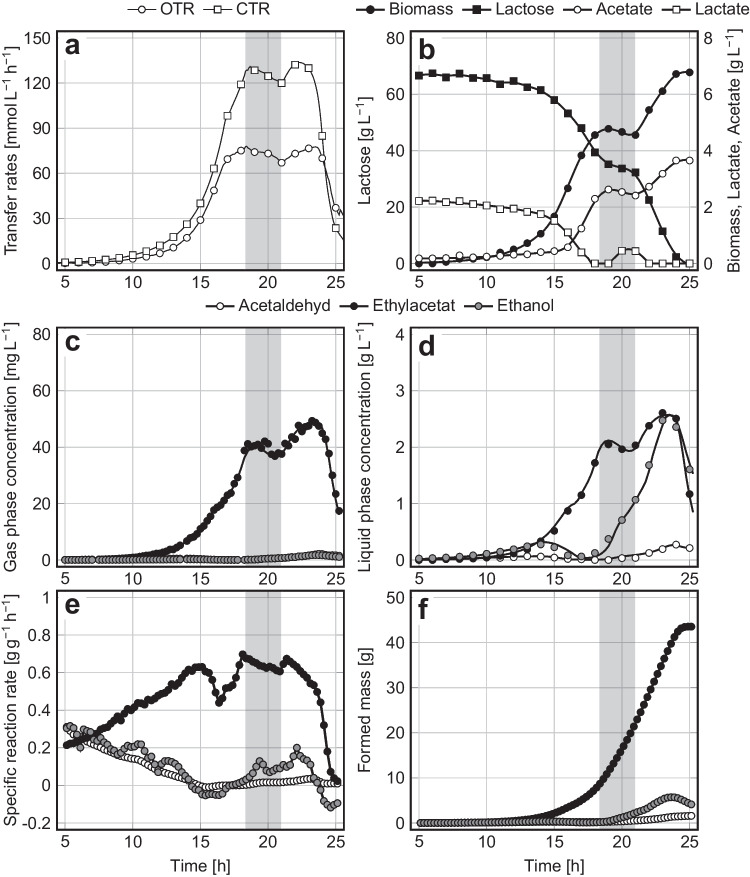
**a **Oxygen transfer rate ($OTR$) and CO_2_ transfer rate ($CTR$); **b **lactose, biomass, acetate, and lactate concentration; **c **gas phase concentrations, **d **liquid phase concentrations, **e **biomass-specific reaction rates, and **f **masses of formed ethyl acetate, ethanol, and acetaldehyde during the aerobic fed-batch cultivation of *K. marxianus* DSM 5422 under iron-limited conditions in a stirred bioreactor using DWP^‒Fe^ medium; Cultivation at 40 °C, aeration with 60 L h^‒1^, and pH 5.1; the process started with 1 L DWP^‒Fe^ medium and was continued by feeding another 1 L DWP^‒Fe^ medium with a rate of 0.37 L h.^‒1^ (marked by the gray area)

The initial batch phase was well comparable with the previously performed pure batch cultivation (compare Figs. 2 and 3). The sugar concentration remained nearly constant during the feeding period, as desired (Fig. 3b). The feeding of fresh medium increased the culture volume from around 1 to 2 L within 2.7 h. The expansion of the liquid volume influenced all variables that are related to the culture volume, such as $\mathrm{OTR}$ and $\mathrm{CTR}$ (Fig. 3a), biomass concentration (Fig. 3b), and dissolved metabolites (Fig. 3d). After completion of the feed, the microbial utilization of the residual sugar continued until its depletion 4 h later (Fig. 3b). The biomass-specific synthesis rate of ethyl acetate remained on a high level during the feeding period and thereafter as well (Fig. 3e). The mass of formed ethyl acetate was nearly twice as high as in the batch process.

Another fed-batch process was performed with the only difference that the pH value was switched from initially 5.1 to 5.3 when the CO_2_ content of the exhaust gas exceeded 0.001 L L^‒1^. The process lasted 24.7 h and ran similar to the process depicted in Fig. 3, showing that high productivity is achieved even at slight pH variation (Online Resource 5).

### Iron-limited repeated-batch cultivation

The harsh conditions in the mineral-rich DWP-based medium, combined with iron limitation, could potentially reduce the viability of the cultivated yeast. However, quantification of the proportion of dead cells in the yeast population during batch cultivations showed that the fraction of living cells was constantly high independently of the applied pH (97.4% living cells on an average at depletion of sugar in batch processes; Table 2). Given that cell viability was high at the end of the (fed-)batch cultures, we investigated whether ethyl acetate production could be extended by a repeated-batch process.

**Fig. 4:**
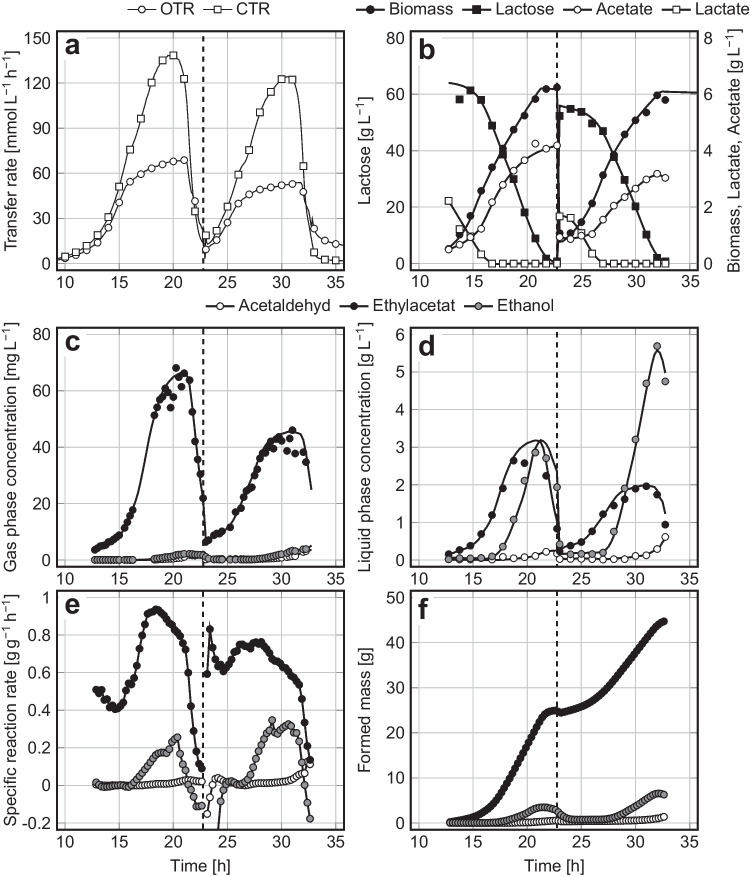
**a **Oxygen transfer rate ($OTR$) and CO_2_ transfer rate ($CTR$); **b **lactose, biomass, acetate, and lactate concentration; **c **gas phase concentrations, **d **liquid phase concentrations, **e **biomass-specific reaction rates, and **f **masses of formed ethyl acetate, ethanol and acetaldehyde during the aerobic repeated-batch cultivation of *K. marxianus* DSM 5422 under iron-limited conditions in a stirred bioreactor using 1 L DWP^‒Fe^ medium; Cultivation at 40 °C, aeration with 60 L h^‒1^, and pH 5.1; after depletion of the sugar at 20.8 h, 0.9 L of cell suspension were replaced by 0.9 L DWP.^‒Fe^ medium (marked by a dashed line)

The repeated batch cultivation was started as a normal batch process with 1 L DWP^‒Fe^ medium (like the process depicted in Fig. 2). Immediately after depletion of the sugar, 0.9 L of the culture broth was replaced by 0.9 L fresh DWP^‒Fe^ medium. Medium exchange was accomplished within 15 min. The initial batch process ran as usual (compare Figs. 4 and 2). No visible stagnation of the process was observed after replacement of 90% of the cell suspension by fresh medium; the OTR, CTR, biomass growth, and ester synthesis were at the expected levels, and the biomass-specific synthesis rate of ethyl acetate was constantly high. Complete utilization of the supplied sugar in the second batch required only 12 h, i.e., the second batch was much shorter compared to the first batch, which is explained by the higher level of initial biomass. The amounts of formed ethyl acetate and ester yields were similar in both stages (24.7 g and 0.38 g g^–1^ vs. 20.3 g and 0.36 g g^–1^). Formation of co-metabolites did not play an important role.

Another repeated batch process at slightly modified pH conditions gave similar results (Online Resources 6).

## Discussion

Delactosed whey permeate (DWP) is a waste stream of the dairy industry with a high content of microbially utilizable sugars (mainly lactose and some galactose). DWP is also characterized by a high load of minerals, which is known to inhibit microbial growth (Maddox 1995; Mahmoud and Kosikowski 1982) and therefore complicates microbial valorization of DWP.

In this work, DWP was used for microbial production of ethyl acetate by the wild-type strain *K. marxianus* DSM 5422. The following detailed investigations were performed: (a) exploration of a suitable dilution of DWP for limiting the inhibitory effect of minerals on growth to a tolerable level, (b) determination of an optimal pH value for conversion of DWP-borne sugar into ethyl acetate with a high yield and rate, and (c) improvement of the process performance by applying fed-batch and repeated-batch cultivations.

### Inhibition of growth by whey-borne minerals

Inhibition of *K. marxianus* in DWP-based media could result from dissolved sugars, minerals, and/or synthesized ethyl acetate. According to Moulin et al. (1980) and Saini et al. (2017), inhibition in whey-borne media is predominantly caused by dissolved minerals rather than lactose. Inhibition of yeasts by ethyl acetate is significant (Inoue et al. 1994; Tabachnick et Joslyn 1953; Urit et al. 2013b) but becomes irrelevant when formed ethyl acetate is quickly removed by stripping from the aerated bioreactor (Löser et al. 2013; Urit et al. 2011, 2013b). Growth inhibition by DWP thus mainly originates from dissolved minerals.

In the studied case, the yeast could even grow in non-diluted DWP (Fig. 1a), but the growth rate was markedly diminished and the lag phase enormously extended, which prolonged the cultivation process by a factor of 4.5 compared to highly diluted DWP (Fig. 1b; for more details, see Online Resource 3). Such a prolongation is very unfavorable from the economic point of view. Therefore, all further experiments were performed with medium based on DWP, which was diluted 1:1 (*v/v*) with water.

### General course of iron-limited batch processes in DWP^‒Fe^ medium

*K. marxianus* in general and *K. marxianus* DSM 5422 in particular can utilize whey-borne lactose and is one of the most potent natural producers of ethyl acetate when cultivated under iron limitation (Hoffmann et al. 2021; Kruis et al. 2018; Löser et al. 2011). In the following, the general course of aerobic batch cultivations of *K. marxianus* DSM 5422 in DWP^‒Fe^ medium will be discussed.

An exemplary experiment, performed at pH 5.1, is depicted in Fig. 2. The yeast grew at first exponentially, which became clearly visible from the $\mathrm{OTR}$ and $\mathrm{CTR}$ (Fig. 2a), but later changed to a linear growth mode (Fig. 2b) due to iron limitation (Online Resource 7). Iron limitation appeared early despite a quite high iron content of 200 µg L^–1^ of the DWP^‒Fe^ medium. An iron-limited metabolism is initiated when the intracellular iron content (${x}_{\mathrm{Fe}}$) falls below a critical value. The ${x}_{Fe}$ value increases due to iron uptake and decreases due to cell expansion at growth: ${\text{d}}{x}_{\mathrm{Fe}}/{\text{d}}t=\psi -\mu \cdot {x}_{\mathrm{Fe}}$; when the $\mu \cdot {x}_{\mathrm{Fe}}$ term exceeds the iron-uptake rate ($\psi$), then the iron content of the cells continuously declines (Löser et al. 2018). A low concentration of iron in the culture medium is the usual reason for a restricted uptake of iron. Here, iron limitation at an early process stage could have been caused by impairment of the iron uptake by medium constituents. Impairment of iron uptake by citrate has already been observed earlier for *K. marxianus* DSM 5422 in another whey-based medium (Löser et al. 2018).

Iron limitation initiated formation of ethyl acetate and some ethanol, acetaldehyde, and acetate (Figs. 2b to f, Table 2). The reason for synthesis of these metabolites has been explained earlier (Hoffmann et al. 2021; Kruis et al. 2018; Löbs et al. 2018; Löser et al. 2015a; Thomas and Dawson 1978). Acetate formation is presumably associated with the thioesterase activity of the Eat1 enzyme which synthesizes ethyl acetate but also hydrolyses acetyl-CoA (Kruis et al. 2017). Acetate is known to inhibit growth of *K. marxianus* DSM 5422 on lactose at lower pH values (Martynova et al. 2016).

The formed metabolites differed in terms of their utilization behavior. Ethyl acetate and acetaldehyde were not degraded; ethanol was utilized quite quickly after sugar depletion, while acetate was metabolized only very slowly. The absent utilization of ethyl acetate by *K. marxianus* DSM 5422 is surprising since Kruis et al. (2017) have found some esterase activity for Eat1, which hydrolyses ethyl acetate to ethanol and acetate. Some strain-dependent variations of Eat1 specificity may thus exist.

Ethyl acetate and acetaldehyde were quickly stripped; ethanol was slowly stripped, while acetate was not stripped at all. Volatile ethyl acetate and acetaldehyde were thus quickly discharged from the bioreactor (Fig. 2c), while less volatile ethanol and non-volatile acetate preferably accumulated in the culture broth (Figs. 2b and d). The lower the volatility of a metabolite is, the smaller the mass ratio between stripped and formed metabolite is. This explains why the selectivity of ester stripping is larger than the selectivity of ester formation (Table 2).

### pH dependency of the iron-limited batch process

The pH significantly influenced biomass formation, product spectrum, product selectivity, and process duration. Although ethyl acetate was the main product in all cultivations, the process at pH 5.1 resulted in the best performance when taking all assessment criteria together: the amount of grown biomass was small, the process was fastest, and the mass of formed ethyl acetate, the ester yield, and the synthesis rate were highest (Table 2). On the other hand, the masses of formed ethanol and acetaldehyde were minimal, causing the best selectivity of ester formation and ester stripping (76.5 and 97.8%, respectively). Acetate was the only co-metabolite that was formed in higher amounts at pH 5.1.

The process at pH 5.4 exhibited the second-best performance. Beyond the pH range from 5.1 to 5.4, the processes became disadvantageous regarding several aspects such as ester yield, synthesis rate, and formation of undesirable co-metabolites (Table 2).

The used DWP medium is complex so that several reasons for the pH impact on yeast growth and product formation are conceivable. These possible reasons will be specifically discussed next.

(a) General pH dependency of yeast growth: Microbial growth clearly depends on the pH. The growth rate and biomass yield of *K. marxianus* are affected at pH values below 3.5 and above 7.0 (Antoce et al. 1997; Löser et al. 2015b; Rouwenhorst et al. 1988; Urit et al. 2013a; Vivier et al. 1993). Here, the pH only varied from 4.8 to 5.9, and an influence was only observed in DWP^–Fe^ but not in DWP^+Fe^ medium (Online Resource 7). Therefore, the pH of the medium alone could not be responsible for the observed pH dependencies.

(b) Effect of citrate: The DWP^‒Fe^ medium contains 9.5 g L^–1^ citrate, which originates from the processed milk. Martynova et al. (2016) have not observed any adverse effect of citrate on growth of *K. marxianus* DSM 5422 in lactose medium containing 7.7 g L^–1^ citrate and surplus iron at pH values ranging from 4 to 6. It is thus unlikely that citrate itself affected the yeast metabolism as observed.

However, the pH could affect the iron uptake by yeasts. The intracellular iron content, ${x}_{\mathrm{Fe}}$, influences the yeast growth and ester synthesis (for details see above). The growth is slowed down and the ester synthesis is intensified when ${x}_{\mathrm{Fe}}$ falls below 30 µg g^‒1^ (Löser et al. 2012). DWP^–Fe^ medium contains 200 µg L^–1^ iron so that complete iron uptake should enable growth of nearly 7 g L^‒1^ biomass without noticeable iron limitation but, in fact, ester synthesis started at a considerably lower cell concentration (Fig. 2). This observation let us assume that uptake of dissolved iron was hampered by medium constituents, most likely by citrate.

It has been shown that citrate impairs complete uptake of iron from the culture medium (Löser et al. 2018). Citrate and iron form chelate complexes. Several iron-citrate complexes exist where the structure of the predominating complex depends on the molar iron/citrate ratio as well as the pH (Evans et al. 2008; Silva et al. 2009). Citrate is a trivalent acid with pK values of 3.13, 4.76, and 6.40 (Goldberg et al. 2002). These pK values are similar to the studied pH values, ranging from 4.8 to 5.9. Variation of the pH thus changes the proportion of the existing iron-citrate complexes (Evans et al. 2008; Silva et al. 2009). It is assumed that the various Fe-citrate complexes differ in their competition with yeast iron transporters. Thus, pH variation causes a changed spectrum of Fe-citrate complexes, which, in turn, affects the iron uptake, followed by modulation of ${x}_{\mathrm{Fe}}$, growth rate and metabolite synthesis. The situation is comparable with the binding of iron by transferrin, which is influenced by citrate and the pH as well (Evans et al. 2008).

It has been shown by Armstrong and Yamazaki (1984) that complexation of iron may initiate ester synthesis during cultivation of *C. jadinii* in iron-rich medium containing ethylenediaminetetraacetic acid (EDTA). EDTA strongly chelates Fe^2+^ and Fe^3+^ ions (Jazimirski et Wassiljew 1963) so that iron is no longer bioavailable. The inhibition of iron uptake by the chelating action of citrate has already been demonstrated for *S. cerevisiae* (Kwok et al. 2006) and marine phytoplankton (Garg et al. 2007; Sudak et al. 2012).

(c) Effect of lactate: DWP^‒Fe^ medium contained 2.5 g L^‒1^ lactate (pK = 3.86 (Lee 2021)), which originates from milk processing by *lactobacilli* and could also play a role in the here-discussed connection. According to Hamada et al. (2005), lactate exhibits some iron-complexing properties which depend on the pH as well. Lactate is consumed by the yeast (Fig. 2b), so the complexation of iron by lactate gradually disappears during the process which could heighten the bioavailability of iron.

These potential impacts on the pH-dependent growth and metabolite synthesis may superimpose each other. It is therefore not possible to definitely specify the one reason.

### Iron-limited fed-batch and repeated-batch processes

Inconvenient cultivation conditions like a high salt load, accumulation of inhibitory products, or a deficit of oxygen can harm microbes, which becomes visible from a declining metabolic activity, a reduced growth rate, or even cell death (Basso et al. 2008). For example, a switch from aerobic to anaerobic conditions during cultivation of *K. marxianus* DSM 5422 in whey-based medium initiated ethanol synthesis, but the missing oxygen let the growth nearly stop and the rate of ethanol synthesis gradually decline (Löser et al. 2015a).

One would expect that the high mineral content of DWP^‒Fe^ medium together with the iron-limited conditions reduce the viability of the yeast. However, cell staining with PI revealed a generally low proportion of dead cells (2.6% dead or 97.4% living cells at the end of batch cultivations on average; Table 2).

A high viability and metabolic activity of grown biomass allow its reuse in fed-batch or repeated-batch processes. Such cell recycling could save sugar for production of biomass, which is required as a biocatalyst for synthesis of the target product, and could reduce cultivation time (Amorim et al. 2011), which make the process economically superior over simple batch processes.

This concept was first confirmed by fed-batch cultivations using 1 L DWP^‒Fe^ medium for the initial batch process and another 1 L DWP^‒Fe^ medium in the feed period (Fig. 3, Online Resource 5). The mass of ethyl acetate formed in the fed-batch processes was twice as high as in the simple batch process. However, the duration of the fed-batch processes was only 3 h longer than the duration of the simple batch process, showing a significant economic effect (doubling the amount of product at only slight prolongation of the process). And there is even more time saved due to a reduced turn-around time for preparation of the process (only one fed-batch process instead of two batches). The formation of by-products in fed-batch cultivations was comparably low as in the batch process, which resulted in similar selectivities of ester synthesis and ester stripping (Table 2). The viability of the biomass at the end of the fed-batch processes was still high, which would allow the addition of more feed. Further development of the process could consist in using non-diluted DWP as a feed since inhibition of yeast growth by DWP-borne minerals is less important in the feeding phase, but the minerals must not inhibit ester synthesis.

Another opportunity for extended use of biomass consists of repeated-batch cultivation. This concept was tested by performing a normal batch process with 1 L DWP^‒Fe^ medium, which was continued as a second batch process by substitution of 0.9 L cell suspension with 0.9 L fresh DWP^‒Fe^ medium (Fig. 4, Online Resource 6). The mass of formed ethyl acetate was almost twice as high as in the simple batch processes. Losses of carbon due to formation of by-products were not higher in the repeated-batch processes in comparison with the batch process. The second cycle of the repeated-batch process was about 11 h shorter than a simple batch process due to the higher initial cell concentration, but the time savings were less distinctive than in the fed-batch process since only part of the formed biomass remained in the bioreactor. In large-scale processes, a higher proportion of biomass could be recycled, e.g., by separation of biomass from the cell suspension via continuous centrifugation, and thus accelerate the process even more. The viability of the biomass was high even at the end of the repeated-batch process, so the continuation of the process by a further cycle seems possible.

In this work, the best results were obtained for the repeated-batch process with the following parameters (Table 2): ${Y}_{\mathrm{EA}/\mathrm{S}}$ = 0.368 g g^-1^, ${Y}_{\mathrm{EA}/\mathrm{S}}$-${Y}_{\mathrm{EA}/\mathrm{S},\mathrm{max}}$ = 71.5%, ${R}_{\mathrm{EA},\mathrm{max}}$ = 4.67 g L^-1^ h^-1^, and ${r}_{\mathrm{EA},\mathrm{max}}$ = 0.94 g g^-1^ h^-1^. A literature survey of ethyl acetate production with wild-type yeasts from sugar (Online Resource 2) found the following maximum values: $Y_{\mathrm{EA}/\mathrm S}$= 0.289 g g^-1^ and $Y_{\mathrm{EA}/\mathrm S}$-$Y_\mathrm{EA/S,max}$ ratio = 56.2% (Urit et al. 2013a), ${R}_{\mathrm{EA},\mathrm{max}}$ = 5.33 g L^-1^ h^-1^ (Urit et al. 2011), and ${r}_{\mathrm{EA},\mathrm{max}}$ = 0.83 g g^-1^ h^-1^ (Urit et al. 2012). This comparison demonstrates that the performance of the DWP-based repeated-batch cultivation outperforms the production metrics of all hitherto published processes.

## Supplementary Information

Below is the link to the electronic supplementary material.

Supplementary file1 (PDF 249 KB)

Supplementary file2 (PDF 447 KB)

Supplementary file3 (PDF 430 KB)

Supplementary file4 (PDF 621 KB)

Supplementary file5 (PDF 625 KB)

Supplementary file6 (PDF 627 KB)

Supplementary file7 (PDF 388 KB)

## Data Availability

All data presented in this study are available from the corresponding author upon reasonable request.
