# Supplementary material for: Utilization of delactosed whey permeate for the synthesis of ethyl acetate with Kluyveromyces marxianus
Source: Appl Microbiol Biotechnol. 2023 Feb 14;107(5-6):1635–48. doi: 10.1007/s00253-023-12419-1 (PMC10006051; doi:10.1007/s00253-023-12419-1)
Supplement: Supplementary file 1 — Supplementary file1 (PDF 249 KB) [file 253_2023_12419_MOESM1_ESM.pdf]

Online Resource 1

Physicochemical properties of delactosed whey permeate (DWP)

Title: Utilization of delactosed whey permeate for the synthesis of ethyl acetate with *Kluyveromyces marxianus*

Journal: Applied Microbiology and Biotechnology

Authors: Andreas Hoffmann [1], Alexander Franz [1,2], Thomas Walther [1], Christian Löser [1]

[1] Chair of Bioprocess Engineering, Institute of Natural Materials Technology, Technische Universität Dresden, 01062 Dresden, Germany

[2] Chair of Biophysical Chemistry, Institute of Biochemistry, University of Leipzig, 04103 Leipzig, Germany

Corresponding author: Dr. habil. Christian Löser (christian-loeser@tu-dresden.de)

Table OR1.1 Physicochemical properties of delactosed whey permeates (DWP). HPLC = High performance liquid chromatography; IC = Ion chromatography; ICP-MS = Inductively coupled plasma mass spectrometry.

Parameter	Unit	Analytical Method	DWP 1[a]	DWP 2	DWP 3
pH	[-]	pH sensor	6.6	5.4	5.5
Density	[g L^{-1}]	mass of a defined volume	1114	1165	1155
Ash	[g L^{-1}]	DIN 10341	47.5	93.8	81.8
Dry matter	[g L^{-1}]	DIN 10348	266.6	366.7	392.1
Lactose	[g L^{-1}]	HPLC	146.0	223.5	219.6
Galactose	[g L^{-1}]	HPLC	4.6	13.3	11.8
Glucose	[g L^{-1}]	HPLC	0.50	1.50	2.40
NO_3^--N	[g L^{-1}]	IC	<0.1	<0.1	<0.1
NH_4^--N	[g L^{-1}]	IC	0.4	1.4	0.9
Urea-N	[g L^{-1}]	(Löser et al. 2015)	0.6	0.6	0.9
Proteins-N	[g L^{-1}]	(Bradford 1976)	0.85	2.32	3.33
Total-N	[g L^{-1}]	DIN EN ISO 8968-1	17.9	33.5	37.5
SO_4^{2-}-S	[g L^{-1}]	IC	1.9	2.9	2.7
Total S	[g L^{-1}]	ICP-MS	0.7	0.4	1.0
PO_4^{3-}-P	[g L^{-1}]	IC	2.0	4.9	3.0
Total P	[g L^{-1}]	ICP-MS	3.5	4.8	4.6
Fluor	[g L^{-1}]	IC	<0.1	<0.1	<0.1
Chlor	[g L^{-1}]	IC	13.2	19.3	19.9
Sodium	[g L^{-1}]	ICP-MS	6.7	2.1	7.6
Potassium	[g L^{-1}]	ICP-MS	16.6	8.7	24.3
Magnesium	[g L^{-1}]	ICP-MS	0.7	0.7	1.0
Calcium	[g L^{-1}]	ICP-MS	0.6	8.1	1.7
Iron	[mg L^{-1}]	ICP-MS	0.4	1.3	0.3
Copper	[mg L^{-1}]	ICP-MS	0.1	0.1	0.9
Zinc	[mg L^{-1}]	ICP-MS	1.2	21.4	0.6
Nickel	[mg L^{-1}]	ICP-MS	<0.1	<0.1	0.1
Succinate	[g L^{-1}]	IC	0.1	0.2	0.2
Malate	[g L^{-1}]	IC	0.4	0.5	0.4
Pyruvate	[g L^{-1}]	IC	2.9	2.8	3.1
Acetate	[g L^{-1}]	IC	0.3	0.6	0.7
KGA	[g L^{-1}]	IC	<0.1	<0.1	<0.1
Fumarate	[g L^{-1}]	IC	0.2	0.3	0.3
Lactate	[g L^{-1}]	HPLC	5.0	11.9	11.1
Citrate	[g L^{-1}]	HPLC	19.0	30.1	25.8

[a] This DWP was used as base for the DWP medium used in the main article

Bradford MM (1976) A rapid and sensitive method for the quantitation of microgram quantities of protein utilizing the principle of protein-dye binding. Anal Biochem 72:248–254. doi: 10.1006/abio.1976.9999

Löser C, Urit T, Gruner E, Bley T (2015) Efficient growth of *Kluyveromyces marxianus* biomass used as a biocatalyst in the sustainable production of ethyl acetate. Energ Sustain Soc 5(2): 1–15. doi: 10.1186/s13705-014-0028-2
