# Supplementary material for: Utilization of delactosed whey permeate for the synthesis of ethyl acetate with Kluyveromyces marxianus
Source: Appl Microbiol Biotechnol. 2023 Feb 14;107(5-6):1635–48. doi: 10.1007/s00253-023-12419-1 (PMC10006051; doi:10.1007/s00253-023-12419-1)
Supplement: Supplementary file 2 — Supplementary file2 (PDF 447 KB) [file 253_2023_12419_MOESM2_ESM.pdf]

Online Resource 2

Yields and rates of microbial ethyl acetate synthesis

Title: Utilization of delactosed whey permeate for the synthesis of ethyl acetate with *Kluyveromyces marxianus*

Journal: Applied Microbiology and Biotechnology

Authors: Andreas Hoffmann [1], Alexander Franz [1,2], Thomas Walther [1], Christian Löser [1]

[1] Chair of Bioprocess Engineering, Institute of Natural Materials Technology, Technische Universität Dresden, 01062 Dresden, Germany

[2] Chair of Biophysical Chemistry, Institute of Biochemistry, University of Leipzig, 04103 Leipzig, Germany

Corresponding author: Dr. habil. Christian Löser (christian-loeser@tu-dresden.de)

Used symbols are listed in the main part of the work or are explained here in the text. References are labelled by [...] and listed at the end of this Online Resource.

Reported yields of ethyl acetate in the literature were already summarized earlier (Table 2 in [1]). Here, this review has been extended in many respects: (1) ester yields are given differentiated for various process conditions; (2) varied parameters for influencing the ester synthesis are indicated; (3) the trigger for inducing the ester synthesis is named (differentiated into intended and unintended induction); (4) the volume-specific as well as the biomass-specific synthesis rates are given; (5) the used cultivation system is characterized; and (6) the experimental procedure is evaluated regarding possible losses of ester during the process.

Restrictions

In this Supporting information, only processes with ethyl acetate synthesis by growing cultures have been evaluated while processes using resting cells (e. g., described in [2–4]) have been ignored. All considered processes used sugar as a substrate, while processes based on ethanol (e. g., in [2–10]) or a mixture of ethanol and sugar (e. g., in [11–14]) were not evaluated. A further restriction concerns the microorganisms; only wild-type strains of yeasts were taken into account while bacteria and genetically engineered yeasts (e. g., in [14–16]) were excluded.

Explanations

Yeast strain The list of ethyl acetate synthesizing microorganisms is restricted to yeasts and to wild-type strains although a growing number of publications deals with the formation of ethyl acetate by genetically modified yeasts and bacteria. The given yeast names are often not identical with the names used in the original reference, i. e., the naming is orientated on the current state of knowledge.

Substrate Synthesis of ethyl acetate has been studied based on diverse sugars and ethanol as well. The data tabulated below are restricted to processes based on sugars. The most frequently used sugar was glucose, but some studies were based on other hexoses such as fructose and galactose. Another often used raw material was whey consisting of lactose and some galactose (here quoted as lactose). Processes with ethanol as a substrate have been excluded from Table OR2.1.

Varied parameter The third column of Table OR2.1 gives information about the process parameter which was variegated in the considered study to take influence on the ester synthesis. However, such a parameter variation only occurred in part of the evaluated references.

Trigger of ester synthesis The synthesis of ethyl acetate in wild-type strains is induced by special cultivation conditions such as limitation of yeast growth by iron, copper and/or oxygen. Alternatively, ester synthesis can also be induced by partial inhibition of the electron-transport chain using specific inhibitors. Induction of ester synthesis occurred intentionally or accidentally. For this reason, it was differentiated between an intended and an unintended induction. For example, there are studies where ester synthesis had been induced by a lack of iron but, at the same time, oxygen became a limiting factor as well (in this case, iron limitation was the intended trigger, while oxygen limitation was the unintended trigger). The risk of oxygen limitation develops at aerobic yeast cultivation in sugar-rich culture media without adequate mixing, e. g., at cultivation in shake flasks. Ethanol synthesis by Crabtree-negative yeasts may refer to oxygen limitation.

$Y_{EA/S}$ This parameter is the yield of ethyl acetate which is calculated from the grams of synthesized ester related to the grams of consumed sugar. The parameter was calculated for the total process or for the moment when the maximum amount of ester had been synthesized (in case the synthesized ester was consumed again by the yeasts). In case of absent sugar data, a complete sugar utilization was assumed. Values given in brackets were not stated directly in the cited literature but were calculated from the published data.

$Y_{EA/S}/Y_{EA/S,max}$ This parameter is the yield of ethyl acetate related to the possible maximum yield, given in percent. This maximum yield is obtained from stoichiometric considerations, when the sugar is assumedly exclusively converted into ethyl acetate without any yeast growth and by-product synthesis. Kruis [S16] named this $Y_{EA/S}$-$Y_{EA/S,max}$ ratio pathway efficiency. The $Y_{EA/S,max}$ value depends on the metabolic pathway and the used substrate. Here, synthesis of ethyl acetate from ethanol and acetyl-CoA via the Eat1 enzyme is assumed, where acetyl-CoA originates from a respiratory process, resulting in the following maximum yields: $Y_{EA/Glucose,max}$ = 0.489 g g^{-1}, $Y_{EA/Lactose,max}$ = 0.515 g g^{-1}, or $Y_{EA/EtOH,max}$ = 0.956 g g^{-1}. Values given in brackets were not stated directly in the cited literature but were calculated from the published data.

$R_{EA,max}$ This is a process parameter describing the maximum volume-specific rate of ester synthesis and is given in grams of formed ester per liter reaction volume and per hour. When this parameter is changing during the considered process, then the maximum value is given. Values in brackets were not stated directly in the cited literature but were calculated from the depicted data.

$r_{EA,max}$ This is a biological parameter describing the maximum biomass-specific synthesis rate of ethyl acetate and is given in grams of formed ester per gram of biomass dry weight and per hour. When this parameter is changing during the considered process, then the maximum value is given. Values in brackets were not stated directly in the cited literature but were calculated from depicted data.

Cultivation system The used cultivation system is shortly characterized. Cultivations have been performed in standing or shaken flasks or in stirred bioreactors. Culture flasks were fitted with cotton plugs, or the culture flasks were completely sealed with rubber plugs or screw cups (then referred as to ‘sealed’). Stirred bioreactors were operated in the batch mode (then simply named ‘bioreactor’) or in a continuous mode (then termed as ‘chemostat’). Cultivation systems were usually aerated, but in some cases purged with N_2 (for anaerobic conditions) or with pure O_2 (for a better oxygen transfer).

Losses of ester? Ethyl acetate is highly volatile and is therefore stripped from flushed cultivation systems (e. g., from aerated bioreactors) or leaves the system by diffusion (e. g., from cotton-plugged shake flasks). Such losses of ethyl acetate during the process have to be taken into account for precise balancing of ester synthesis. The entry ‘yes’ means,

that such a loss occurred without adequate consideration, while ‘no’ stands for processes in completely sealed systems without such losses or for losses which were quantified. The term ‘probably’ labels processes where a loss of ester seems plausible. The superscripts give more specified information on this subject.

Reference The references are given in form of [...] and are listed at the end of this Supporting information.

Table OR2.1 Yields and rates of ester synthesis from the literature depending on the cultivated strain and the cultivation conditions (detailed explanation above).

**Yeast species, Strain**	**Substrate**	**Varied parameter**	**Trigger of ester synthesis**		$Y_{EA/S}$ [g_{EA} g_S^{-1}]	$Y_{EA/S}/Y_{EA/S,max}$ [%]	$R_{EA,max}$ [g_{EA} $L^{-1}h^{-1}$]	$r_{EA,max}$ [g_{EA} $g_X^{-1}h^{-1}$]	**Cultivation system**	**Losses of ester**	**Reference**
			**intended**	**unintended**							
Cyberlindnera fabianii CBS 5640	Glucose	Fe availability	Fe limitation	-	0.16	32.7	(0.82)	(0.26)	Aerated chemostat	no [a)]	[17]
Cyberlindnera fabianii CBS 5640	Glucose	Fe availability	-	-	0.001	0.2	-	-	Aerated chemostat	no [a)]	[17]
Cyberlindnera jadinii ATCC 9950	Glucose	O_2 availability	Fe limitation	O_2 limitation	(0.09)	(18)	(0.79)	-	Shaken sealed flask	no [b)]	[3]
Cyberlindnera jadinii ATCC 9950	Glucose	O_2 availability	Fe + O_2 limitation	-	(0.03 - 0.18)	(6 - 37)	-	-	Shaken flask	probably [c)]	[3]
Cyberlindnera jadinii ATCC 9950	Glucose	Fe availability	heavy Fe limitation	O_2 limitation	(0.25 - 0.26)	(51 - 53)	-	-	Shaken sealed flask	no [b)]	[18]
Cyberlindnera jadinii ATCC 9950	Glucose	Fe availability	medium Fe limitation	O_2 limitation	(0.09)	(18)	-	-	Shaken sealed flask	no [b)]	[18]
Cyberlindnera jadinii ATCC 9950	Glucose	Fe availability	slight Fe limitation	O_2 limitation	(0.04)	(8)	-	-	Shaken sealed flask	no [b)]	[18]
Cyberlindnera jadinii ATCC 9950	Glucose	Fe availability	Fe limitation	O_2 limitation	(0.24)	(49)	(0.12)	-	Shaken sealed flask	no [b)]	[18]
Cyberlindnera jadinii ATCC 9950	Glucose	Fe availability	-	O_2 limitation	(0.00)	(0)	-	-	Shaken sealed flask	no [b)]	[18]
Cyberlindnera jadinii ATCC 9950	Glucose	-	Fe limitation	? O_2 limitation	(0.27)	(56)	(0.23)	(0.15)	Aerated system	yes [d)]	[19]
Cyberlindnera jadinii CECT 1956	Glucose	Fe availability	Fe limitation	-	(0.105)	(21.5)	(0.59)	(0.07)	Aerated chemostat	no [a)]	[17]
Cyberlindnera jadinii CECT 1956	Glucose	Fe availability	-	-	0.000	0.0	-	-	Aerated chemostat	no [a)]	[17]
Cyberlindnera jadinii DSM 2361	Glucose	Limiting factor	-	-	0.000	0.0	0.00	0.000	Aerated bioreactor	no [a)]	[34]
Cyberlindnera jadinii DSM 2361	Glucose	Limiting factor	Fe limitation	-	0.028	5.7	0.11	0.034	Aerated bioreactor	no [a)]	[34]
Cyberlindnera jadinii DSM 2361	Glucose	Limiting factor	O_2 limitation	-	0.021	4.3	0.45	0.053	Aerated bioreactor	no [a)]	[34]
Kluyveromyces lactis CBS 739	Lactose	Culture system	Fe limitation	O_2 limitation	(0.19)	(36)	-	-	Shaken sealed flask	no [b)]	[20]
Kluyveromyces lactis CBS 739	Lactose	Culture system	Fe limitation	O_2 limitation	0.18	35	0.68	-	O_2-purged flask	no a)	[20]
Kluyveromyces lactis CBS 743	Lactose	Culture system	Fe limitation	O_2 limitation	(0.14)	(28)	-	-	Shaken sealed flask	no [b)]	[20]
Kluyveromyces lactis CBS 743	Lactose	Culture system	Fe limitation	O_2 limitation	0.18	35	0.59	-	O_2-purged flask	no [a)]	[20]
Kluyveromyces lactis CBS 2359	Glucose	Fe availability	Fe limitation	-	0.04	8.2	(0.23)	(0.046)	Aerated chemostat	no [a)]	[17]
Kluyveromyces lactis CBS 2359	Glucose	Fe availability	-	-	0.000	0.0	-	-	Aerated chemostat	no [a)]	[17]
Kluyveromyces marxianus	Lactose	O_2 availability	Fe limitation	-	(0.013)	(2.6)	-	-	Aerated bioreactor	yes [d)]	[12]
Kluyveromyces marxianus	Lactose	O_2 availability	Fe limitation	-	(0.019)	(3.6)	(0.02)	-	Aerated bioreactor	yes [d)]	[12]
Kluyveromyces marxianus	Lactose	O_2 availability	Fe + O_2 limitation	-	(0.004)	(0.8)	(0.02)	-	Aerated chemostat	yes [d)]	[12]
Kluyveromyces marxianus	Lactose	O_2 availability	Fe limitation	-	(0.039)	(7.6)	(0.20)	-	Aerated chemostat	yes [d)]	[12]
Kluyveromyces marxianus	Lactose	C/N ratio	Fe limitation	-	(0.01 - 0.08)	(2.4 - 14.5)	-	-	Aerated chemostat	yes [d)]	[12]
Kluyveromyces marxianus	Lactose	-	Fe + O_2 limitation	-	(0.007)	(1.4)	(0.02)	(0.008)	Aerated chemostat	yes [d)]	[13]
Kluyveromyces marxianus (21 strains)	Lactose	Strain	Fe limitation	O_2 limitation	(0.01 - 0.17)	(2 - 33)	-	-	Shaken sealed flask	no [b)]	[20]
Kluyveromyces marxianus (10 strains)	Lactose	Strain	Fe limitation	O_2 limitation	0.13 - 0.23	25 - 45	0.37 - 0.91	-	O_2-purged flask	no [a)]	[20]
Kluyveromyces marxianus CBS 6556	Glucose	Sugar + strain	-	? O_2 limitation	(0.245)	(50.1)	-	-	Shaken flask	probably [c)]	[15]
Kluyveromyces marxianus CBS 6556	Fructose	Sugar + strain	-	? O_2 limitation	(0.185)	(37.8)	-	-	Shaken flask	probably [c)]	[15]
Kluyveromyces marxianus CBS 6556	Galactose	Sugar + strain	-	? O_2 limitation	(0.190)	(38.9)	-	-	Shaken flask	probably [c)]	[15]
Kluyveromyces marxianus CBS 6556	Glucose	-	Fe limitation	-	(0.186)	(38.0)	0.80	-	Aerated bioreactor	no [a)]	[21]

Table OR2.1 Continuation 1.

Yeast species, Strain	Substrate	Varied parameter	Trigger of ester synthesis		$Y_{EA/S}$ [g_{EA} g_S^{-1}]	$Y_{EA/S}/Y_{EA/S,max}$ [%]	$R_{EA,max}$ [g_{EA} $L^{-1}h^{-1}$]	$r_{EA,max}$ [g_{EA} $g_X^{-1}h^{-1}$]	Cultivation system	Losses of ester	Reference
			intended	unintended							
Kluyveromyces marxianus DSM 5422	Lactose	Medium + Fe avail.	-	-	< 0.01	< 2	-	-	Aerated bioreactor	no a)	[22]
Kluyveromyces marxianus DSM 5422	Lactose	Medium + Fe avail.	Fe limitation	-	0.25	49	5.33	(0.52)	Aerated bioreactor	no a)	[22]
Kluyveromyces marxianus DSM 5422	Lactose	Medium + Fe avail.	-	-	< 0.01	< 2	-	-	Aerated bioreactor	no a)	[22]
Kluyveromyces marxianus DSM 5422	Lactose	Medium + Fe avail.	Fe limitation	-	0.23	45	2.94	-	Aerated bioreactor	no a)	[22]
Kluyveromyces marxianus DSM 5422	Lactose	Medium + Fe avail.	-	-	< 0.01	< 2	-	-	Aerated bioreactor	no a)	[22]
Kluyveromyces marxianus DSM 5422	Lactose	Medium + Fe avail.	Fe limitation	-	0.07	14	0.86	-	Aerated bioreactor	no a)	[22]
Kluyveromyces marxianus DSM 5422	Lactose	Medium + Fe avail.	-	-	< 0.01	< 2	-	-	Aerated bioreactor	no a)	[22]
Kluyveromyces marxianus DSM 5422	Lactose	Medium + Fe avail.	Fe limitation	-	0.19	37	1.69	-	Aerated bioreactor	no a)	[22]
Kluyveromyces marxianus DSM 5422	Lactose	Limiting factor	Fe limitation	-	0.25	49	2.70	0.83	Aerated bioreactor	no a)	[23]
Kluyveromyces marxianus DSM 5422	Lactose	Limiting factor	Cu limitation	-	0.05	9	0.98	0.09	Aerated bioreactor	no a)	[23]
Kluyveromyces marxianus DSM 5422	Lactose	Limiting factor	Zn limitation	-	0.01	2	0.27	0.04	Aerated bioreactor	no a)	[23]
Kluyveromyces marxianus DSM 5422	Lactose	Fe availability	± Fe limitation	-	0.00 - 0.23	0 - 45	-	-	Sealed shaken flask	no b)	[24]
Kluyveromyces marxianus DSM 5422	Lactose	Dilution rate	-	-	0.01 - 0.05	2 - 10	(0.01 - 0.56)	0.000 - 0.031	Aerated chemostat	no a)	[24]
Kluyveromyces marxianus DSM 5422	Lactose	Dilution rate	Fe limitation	-	0.05 - 0.15	10 - 29	(0.25 - 0.49)	0.084 - 0.215	Aerated chemostat	no a)	[24]
Kluyveromyces marxianus DSM 5422	Lactose	Fe availability	± Fe limitation	-	0.05 - 0.22	9 - 43	(0.20 - 1.51)	0.020 - 0.437	Aerated chemostat	no a)	[24]
Kluyveromyces marxianus DSM 5422	Lactose	Temperature	Fe limitation	-	0.198	38.4	1.61	0.54	Aerated bioreactor	no a)	[25]
Kluyveromyces marxianus DSM 5422	Lactose	Temperature	(Fe limitation)	-	0.002	0.4	0.05	0.01	Aerated bioreactor	no a)	[25]
Kluyveromyces marxianus DSM 5422	Lactose	Temperature	Fe limitation	-	0.289	56.2	4.23	0.67	Aerated bioreactor	no a)	[25]
Kluyveromyces marxianus DSM 5422	Lactose	Gas flow	Fe limitation	-	0.213 - 0.219	41.4 - 42.5	2.49 - 2.95	0.26 - 0.40	Aerated bioreactor	no a)	[26]
Kluyveromyces marxianus DSM 5422	Lactose	Fe availability	heavy Fe limitation	-	0.265	51.4	2.45	0.490	Aerated bioreactor	no a)	[27]
Kluyveromyces marxianus DSM 5422	Lactose	Fe availability	medium Fe limitation	-	0.239	46.3	3.13	0.337	Aerated bioreactor	no a)	[27]
Kluyveromyces marxianus DSM 5422	Lactose	Fe availability	slight Fe limitation	-	0.113	21.9	1.95	0.174	Aerated bioreactor	no a)	[27]
Kluyveromyces marxianus DSM 5422	Lactose	O_2 availability	O_2 limitation	-	0.004	0.8	0.05	(0.008)	Aerated bioreactor	no a)	[28]
Kluyveromyces marxianus DSM 5422	Lactose	O_2 availability	O_2 excluded	-	0.001	0.2	0.005	(0.002)	N_2-purged bioreactor	no a)	[28]
Kluyveromyces marxianus DSM 5422	Lactose	ETC inhibitor	± Carboxin	-	0.000 - 0.136	0.0 - 26.4	-	-	Sealed shaken flask	no b)	[28]
Kluyveromyces marxianus DSM 5422	Lactose	ETC inhibitor	± Antimycin A	-	0.000 - 0.037	0.0 - 7.2	-	-	Sealed shaken flask	no b)	[28]
Kluyveromyces marxianus DSM 5422	Lactose	ETC inhibitor	± Cyanide	-	0.001 - 0.098	0.2 -19.0	(0.00 - 0.07)	(0.00 - 0.17)	Sealed shaken flask	no b)	[28]
Kluyveromyces marxianus DSM 5422	Glucose	Sugar + strain	-	? O_2 limitation	(0.188)	(38.4)	-	-	Shaken flask	probably c)	[15]
Kluyveromyces marxianus DSM 5422	Fructose	Sugar + strain	-	? O_2 limitation	(0.170)	(34.8)	-	-	Shaken flask	probably c)	[15]
Kluyveromyces marxianus DSM 5422	Galactose	Sugar + strain	-	? O_2 limitation	(0.218)	(44.7)	-	-	Shaken flask	probably c)	[15]

Table OR2.1 Continuation 2.

Yeast species, Strain	Substrate	Varied parameter	Trigger of ester synthesis		$Y_{EA/S}$ [g_{EA} g_S^{-1}]	$Y_{EA/S}/Y_{EA/S,max}$ [%]	$R_{EA,max}$ [g_{EA} $L^{-1}h^{-1}$]	$r_{EA,max}$ [g_{EA} $g_X^{-1}h^{-1}$]	Cultivation system	Losses of ester	Reference
			intended	unintended							
Kluyveromyces marxianus DSM 5422	Glucose	Fe availability	Fe limitation	-	(0.136)	(27.8)	(1.08)	(0.157)	Aerated chemostat	no a)	[17]
Kluyveromyces marxianus DSM 5422	Glucose	Fe availability	-	-	0.000	0.0	-	-	Aerated chemostat	no a)	[17]
Kluyveromyces marxianus DSM 5422	Glucose	Limiting factor	-	-	0.024	4.9	0.18	0.038	Aerated bioreactor	no a)	[34]
Kluyveromyces marxianus DSM 5422	Glucose	Limiting factor	Fe limitation	-	0.182	37.2	0.86	0.502	Aerated bioreactor	no a)	[34]
Kluyveromyces marxianus DSM 5422	Glucose	Limiting factor	O_2 limitation	-	0.042	8.6	0.56	0.145	Aerated bioreactor	no a)	[34]
Kluyveromyces marxianus DSM 5422	Lactose	Fe availability	-	-	0.009	1.7	0.33	0.05	Aerated bioreactor	no a)	this work
Kluyveromyces marxianus DSM 5422	Lactose	pH value 4.8	Fe limitation	-	0.245	47.5	4.24	0.70	Aerated bioreactor	no a)	this work
Kluyveromyces marxianus DSM 5422	Lactose	pH value 5.1	Fe limitation	-	0.347	67.4	4.65	0.93	Aerated bioreactor	no a)	this work
Kluyveromyces marxianus DSM 5422	Lactose	pH value 5.4	Fe limitation	-	0.302	58.6	4.38	0.95	Aerated bioreactor	no a)	this work
Kluyveromyces marxianus DSM 5422	Lactose	pH value 5.65	Fe limitation	-	0.327	63.5	3.99	0.63	Aerated bioreactor	no a)	this work
Kluyveromyces marxianus DSM 5422	Lactose	pH value 5.9	Fe limitation	-	0.255	49.5	4.31	0.50	Aerated bioreactor	no a)	this work
Kluyveromyces marxianus DSM 5422	Lactose	fed-batch mode	Fe limitation	-	0.311	60.4	3.47	0.70	Aerated bioreactor	no a)	this work
Kluyveromyces marxianus DSM 5422	Lactose	repeated-batch m.	Fe limitation	-	0.368	71.5	4.67	0.94	Aerated bioreactor	no a)	this work
Kluyveromyces marxianus NCYC 143	Glucose	Fe + O_2 availability	heavy Fe limitation	? O_2 limitation	(0.015)	(3.0)	-	-	Shaken flask	probably c)	[8]
Kluyveromyces marxianus NCYC 143	Glucose	Fe + O_2 availability	-	O_2 limitation	(0.0015)	(0.3)	-	-	Shaken flask	probably c)	[8]
Kluyveromyces marxianus NCYC 143	Glucose	Fe + O_2 availability	O_2 excluded	-	0.00	0.0	-	-	Sealed shaken flask	no b)	[8]
Lachancea kluyveri Y708	Glucose	-	-	-	0.080	16.3	(0.64)	(0.117)	Aerated bioreactor	no a)	[29]
Wickerhamomyces anomalus	Glucose	-	-	heavy O_2 limitation	(0.07)	(14)	(0.10)	-	Standing flask	probably c)	[30]
Wickerhamomyces anomalus	Glucose	O_2 availability	-	heavy O_2 limitation	0.123	25.1	-	-	Sealed standing flask	no b)	[31]
Wickerhamomyces anomalus	Glucose	O_2 availability	O_2 excluded	-	0.005	1.1	-	-	Sealed standing flask	no b)	[31]
Wickerhamomyces anomalus	Glucose	growth factors	-	heavy O_2 limitation	(0.13- 0.18)	(27 - 38)	(0.00 - 0.06)	-	Standing flask	probably c)	[5]
Wickerhamomyces anomalus	Glucose	growth factors	-	slight O_2 limitation	(0.074)	(15)	(0.09)	(0.041)	Shaken flask	probably c)	[5]
Wickerhamomyces anomalus CECT 10590	Glucose	O_2 availability	heavy O_2 limitation	-	(0.017)	(3.4)	-	-	Standing flask	probably c)	[32]
Wickerhamomyces anomalus CECT 10590	Glucose	O_2 availability	-	O_2 limitation	(0.104)	(21.2)	-	-	Shaken flask	probably c)	[32]
Wickerhamomyces anomalus DSM 6766	Glucose	O_2 availability	-	-	0	0	0	0	Aerated chemostat	no a)	[14]
Wickerhamomyces anomalus DSM 6766	Glucose	O_2 availability	O_2 limitation	-	0.064	13.1	0.064	-	Aerated chemostat	no a)	[14]
Wickerhamomyces anomalus DSM 6766	Glucose	Fe availability	Fe limitation	-	0.17	34.8	(1.16)	(0.276)	Aerated chemostat	no a)	[17]
Wickerhamomyces anomalus DSM 6766	Glucose	Fe availability	-	-	0.000	0.0	-	-	Aerated chemostat	no a)	[17]
Wickerhamomyces anomalus DSM 6766	Glucose	Limiting factor	-	-	0.000	0.0	0.00	0.000	Aerated bioreactor	no a)	[34]
Wickerhamomyces anomalus DSM 6766	Glucose	Limiting factor	Fe limitation	-	0.046	9.4	0.16	0.044	Aerated bioreactor	no a)	[34]
Wickerhamomyces anomalus DSM 6766	Glucose	Limiting factor	O_2 limitation	-	0.053	10.8	0.63	0.112	Aerated bioreactor	no a)	[34]

Table OR2.1 Continuation 3.

**Yeast species, Strain**	**Substrate**	**Varied parameter**	**Trigger of ester synthesis**		$Y_{EA/S}$ [g_{EA} g_S^{-1}]	$Y_{EA/S}/Y_{EA/S,max}$ [%]	$R_{EA,max}$ [g_{EA} $L^{-1}h^{-1}$]	$r_{EA,max}$ [g_{EA} $g_X^{-1}h^{-1}$]	**Cultivation system**	**Losses of ester**	**Reference**
			**intended**	**unintended**							
Wickerhamomyces anomalus CBS 1984	Glucose	O_2 availability	-	-	-	-	-	0.00005	Aerated bioreactor	yes [d)]	[33]
Wickerhamomyces anomalus CBS 1984	Glucose	O_2 availability	O_2 limitation	-	-	-	-	0.001	Aerated bioreactor	yes [d)]	[33]
Wickerhamomyces ciferrii CBS 111	Glucose	-	Fe limitation	O_2 limitation	(0.130)	(26.5)	(0.16)	-	Shaken flask	probably [c)]	[14]

[a)] = stripping handled adequately, e.g., by analyzing the stripped ester in the exhaust gas
[b)] = stripping not occurring due to cultivation in a completely sealed system
[c)] = assumed losses because of uncertain sealing (e.g., by cotton stoppers) or non-reported sealing
[d)] = stripping handled in a wrong way (e.g., by using an exhaust-gas condenser for retention of stripped ester)

References

[1] Löser C, Urit T, Bley T (2014) Perspectives for the biotechnological production of ethyl acetate by yeasts. Appl Microbiol Biotechnol 98:5397–5415.

[2] Tabachnick J, Joslyn MA (1953) Formation of esters by yeast. II. Investigations with cellular suspensions of *Hansenula anomala*. Plant Physiol 28:681–692.

[3] Armstrong DW, Martin SM, Yamazaki H (1984) Production of ethyl acetate from dilute ethanol solutions by *Candida utilis*. Biotechnol Bioeng 26:1038–1041.

[4] Armstrong DW, Martin SM, Yamazaki H (1984) Production of acetaldehyde from ethanol by *Candida utilis*. Biotechnol Lett 6:183–188.

[5] Tabachnick J, Joslyn MA (1953) Formation of esters by yeast. I. The production of ethyl acetate by standing surface cultures of *Hansenula anomala*. J Bacteriol 65:1–9.

[6] Armstrong DW (1986) Selective production of ethyl acetate by *Candida utilis*. ACS Symp 317:254–265.

[7] Bol J, Knol W, ten Brik B (1987) Optimization of the production of ethyl acetate from ethanol by *Hansenula anomala*. Dechema Monographs 105:235–236.

[8] Willetts A (1989) Ester formation from ethanol by *Candida pseudotropicalis*. Antonie van Leeuwenhoek 56:175–180.

[9] Corzo G, Revah S, Christen P (1995) Effect of oxygen on the ethyl acetate production from continuous ethanol stream by *Candida utilis* in submerged cultures. Develop Food Sci 37B:1141–1154.

[10] Christen P, Domenech F, Páca J, Revah S (1999) Evaluation of four *Candida utilis* strains for biomass, acetic acid and ethyl acetate production from ethanol. Bioresour Technol 68:193–195.

[11] Yong FM, Lee KH, Wong HA (1981) The production of ethyl acetate by soy yeast *Saccharomyces rouxii* NRRL Y-1096. J Food Technol 16:177–184.

[12] Kallel-Mhiri H, Engasser JM, Miclo A (1993) Continuous ethyl acetate production by *Kluyveromyces fragilis* on whey permeate. Appl Microbiol Biotechnol 40:201–205.

[13] Kallel-Mhiri H, Miclo A (1993) Mechanism of ethyl acetate synthesis by *Kluyveromyces fragilis*. FEMS Microbiol Lett 111:207–212.

[14] Kruis AJ, Levisson M, Mars AE, van der Ploeg M, et al. (2017) Ethyl acetate production by the elusive alcohol acetyltransferase from yeast. Metab Eng 41:92–101.

[15] Löbs A-K, Lin J-L, Cook M, Wheeldon I (2016) High throughput, colorimetric screening of microbial ester biosynthesis reveals high ethyl acetate production from *Kluyveromyces marxianus* on C5, C6, and C12 carbon sources. Biotechnol J 11:1–8.

[16] Kruis AJ (2018) Towards biobased ethyl acetate and beyond. Identification and application of the elusive Eat1 enzyme. Dissertation, Wageningen University, Wageningen, the Netherlands.

[17] Kruis AJ, Mars AE, Kengen SWM, Borst JW, et al. (2018) Alcohol acetyltransferase Eat1 is located in yeast mitochondria. Appl Environ Microbiol 84:e01640-18:1–11.

[18] Armstrong DW, Yamazaki H (1984) Effect of iron and EDTA on ethyl acetate accumulation in *Candida utilis*. Biotechnol Lett 6:819–824.

[19] Williams RE, Armstrong DW, Murray WD, Welsh FW (1988) Enzyme and whole cell production of flavor and fragrance compounds. Ann New York Acad Sci 542:406–412.

[20] Löser C, Urit T, Nehl F, Bley T (2011) Screening of *Kluyveromyces* strains for the production of ethyl acetate: design and evaluation of a cultivation system. Eng Life Sci 11:369–381.

[21] Löbs A-K, Engel R, Schwartz C, Flores A, Wheeldon I (2017) CRISPR-Cas9-enabled genetic disruptions for understanding ethanol and ethyl acetate biosynthesis in *Kluyveromyces marxianus*. *Biotechnol.* Biofuels 10:164:1–14.

[22] Urit T, Löser C, Wunderlich M, Bley T (2011) Formation of ethyl acetate by *Kluyveromyces marxianus* on whey: studies of the ester stripping. Bioprocess Biosyst Eng 34:547–559.

[23] Urit T, Stukert A, Bley T, Löser C (2012) Formation of ethyl acetate by *Kluyveromyces marxianus* on whey during aerobic batch cultivation at specific trace-element limitation. Appl Microbiol Biotechnol 96:1313–1323.

[24] Löser C, Urit T, Förster S, Stukert A, Bley T (2012) Formation of ethyl acetate by *Kluyveromyces marxianus* on whey during aerobic batch and chemostat cultivation at iron limitation. Appl Microbiol Biotechnol 96:685–696.

[25] Urit T, Li M, Bley T, Löser C (2013) Growth of *Kluyveromyces marxianus* and formation of ethyl acetate depending on temperature. Appl Microbiol Biotechnol 97:10359–10371.

[26] Urit T, Manthey R, Bley T, Löser C (2013) Formation of ethyl acetate by *Kluyveromyces marxianus* on whey: influence of aeration and inhibition of yeast growth by ethyl acetate. Eng Life Sci 13:247–260.

[27] Löser C, Urit T, Stukert A, Bley T (2013) Formation of ethyl acetate from whey by *Kluyveromyces marxianus* on a pilot scale. J Biotechnol 163:17–23.

[28] Löser C, Urit T, Keil P, Bley T (2015) Studies on the mechanism of synthesis of ethyl acetate in *Kluyveromyces marxianus* DSM 5422. Appl Microbiol Biotechnol 99:1131–1144.

[29] Møller K, Christensen B, Förster J, Piškur J, et al. (2002) Aerobic glucose metabolism of *Saccharomyces kluyveri*: growth, metabolite production, and quantification of metabolic fluxes. Biotechnol Bioeng 77:186–193.

[30] Gray WD (1949) Initial studies on the metabolism of *Hansenula anomala* (Hansen) Sydow. Am J Bot 36:475–480.

[31] Davies R, Falkiner EA, Wilkinson JF, Peel JL (1951) Ester formation by yeasts 1. Ethyl acetate formation by *Hansenula* species. Biochem J 49:58–61.

[32] Rojas V, Gil JV, Piñaga F, Manzanares P (2001) Studies on acetate ester production by non-*Saccharomyces* wine yeasts. Int J Food Microbiol 70:283–289.

[33] Fredlund E, Blank LM, Schnürer J, Sauer U, Passoth V (2004) Oxygen- and glucose-dependent regulation of central carbon metabolism in *Pichia anomala*. Appl Environ Microbiol 70:5905–5911.

[34] Hoffmann A, Kupsch C, Walther T, Löser C (2021) Synthesis of ethyl acetate from glucose by *Kluyveromyces marxianus*, *Cyberlindnera jadinii* and *Wickerhamomyces anomalus* depending on the induction mode. Eng Life Sci 21:154–168.
