# Supplementary material for: Utilization of delactosed whey permeate for the synthesis of ethyl acetate with Kluyveromyces marxianus
Source: Appl Microbiol Biotechnol. 2023 Feb 14;107(5-6):1635–48. doi: 10.1007/s00253-023-12419-1 (PMC10006051; doi:10.1007/s00253-023-12419-1)
Supplement: Supplementary file 3 — Supplementary file3 (PDF 430 KB) [file 253_2023_12419_MOESM3_ESM.pdf]

Online Resource 3

Determination of the specific growth rate (μ) and the lag period (t_{lag})

Title: Utilization of delactosed whey permeate for the synthesis of ethyl acetate with *Kluyveromyces marxianus*

Journal: Applied Microbiology and Biotechnology

Authors: Andreas Hoffmann [1], Alexander Franz [1,2], Thomas Walther [1], Christian Löser [1]

[1] Chair of Bioprocess Engineering, Institute of Natural Materials Technology, Technische Universität Dresden, 01062 Dresden, Germany

[2] Chair of Biophysical Chemistry, Institute of Biochemistry, University of Leipzig, 04103 Leipzig, Germany

Corresponding author: Dr. habil. Christian Löser (christian-loeser@tu-dresden.de)

Methodology

The Inhibition of growth of the yeast *K. marxianus* DSM 5422 by whey-borne minerals was studied during aerobic batch cultivations in whey-borne media. The used media were based on delactosed whey permeate (DWP) which is a waste of milk processing. The DWP is rich in minerals which hold the risk of inhibiting the cultivated yeasts. The DWP was differently diluted with water and supplemented with urea and trace-element solution with iron. Each growth experiment was performed at 40 °C, an aeration rate of 180 L h^{-1} (standard conditions) and a pH value of 5.1. The microbial activity of the yeasts were followed by measuring the CO_2 content in the exhaust gas of the bioreactor.

Further processing of the exhaust gas data has been described in detail by Löser et al. (2021). The exhaust-gas data (i.e., the time-dependent O_2 and CO_2 content of the exhaust gas) allowed to calculate the gas flow of the exhaust gas and thus balancing der CO_2 formation. This data processing delivered the molar flow of microbially formed CO_2 depending on time, $\dot{n}_{CO2}(t)$, given in moles formed CO_2 per hour. This flow of formed CO_2 corresponds to the growth of biomass with the CO_2 yield, $Y_{CO2/X}$, as the proportionality factor:

$$\dot{n}_{CO2}(t) = V_L \cdot Y_{CO2/X} \cdot \frac{dC_X}{dt} \qquad \text{(OR3.1)}$$

Integration gives:

$$\int_0^t \dot{n}_{CO2}(t)\, dt = V_L \cdot Y_{CO2/X} \cdot \int_{C_X(t=0)}^{C_X(t)} dC_X \qquad \text{(OR3.2)}$$

Execution of the integration and rearrangement gives ($n_{CO2}(t{=}0)$ is zero since no CO_2 has been formed at the start of cultivation):

$$C_X(t) = C_X(t{=}0) + \frac{n_{CO2}(t)}{V_L \cdot Y_{CO2/X}} \qquad \text{(OR3.3)}$$

Herein, $C_X(t{=}0)$ is the initial biomass concentration resulting from inoculation, V_L is the volume of the culture medium, and $n_{CO2}(t)$ is the cumulative amount of CO_2 which was formed during yeast growth till the process time t. The inoculated biomass corresponds to a specific amount of CO_2 which was formed during the growth of the inoculated biomass:

$$C_X(t{=}0) = \frac{n_{CO2}(Inoc.)}{V_L \cdot Y_{CO2/X}} \qquad \text{(OR3.4)}$$

Combination of the Equations (OR3.3) and (OR3.4) results in:

$$C_X(t) = \frac{n_{CO2}(Inoc.)}{V_L \cdot Y_{CO2/X}} + \frac{n_{CO2}(t)}{V_L \cdot Y_{CO2/X}} \quad \text{(OR3.5)}$$

Provided that the CO_2 yield, $Y_{CO2/X}$, and the volume of the cultivation medium, V_L, do not change during the process, one yields:

$$C_X(t) \sim n_{CO2}(Inoc.) + n_{CO2}(t) = \Sigma n_{CO2}(t) \quad \text{(OR3.6)}$$

This means that the growth curve strictly correlates with the sum of $n_{CO2}(t)$ and $n_{CO2}(Inoc.)$ so that growth-characterizing parameters can also be derived from the $n_{CO2}(t)$ curve.

In Figure OR3.1, the amount of cumulatively formed CO_2 depending on the process time including the CO_2 released at formation of the inoculum ($\Sigma n_{CO2}(t)$) is shown as a logarithmic plot for a typical cultivation process based on 75% DWP.

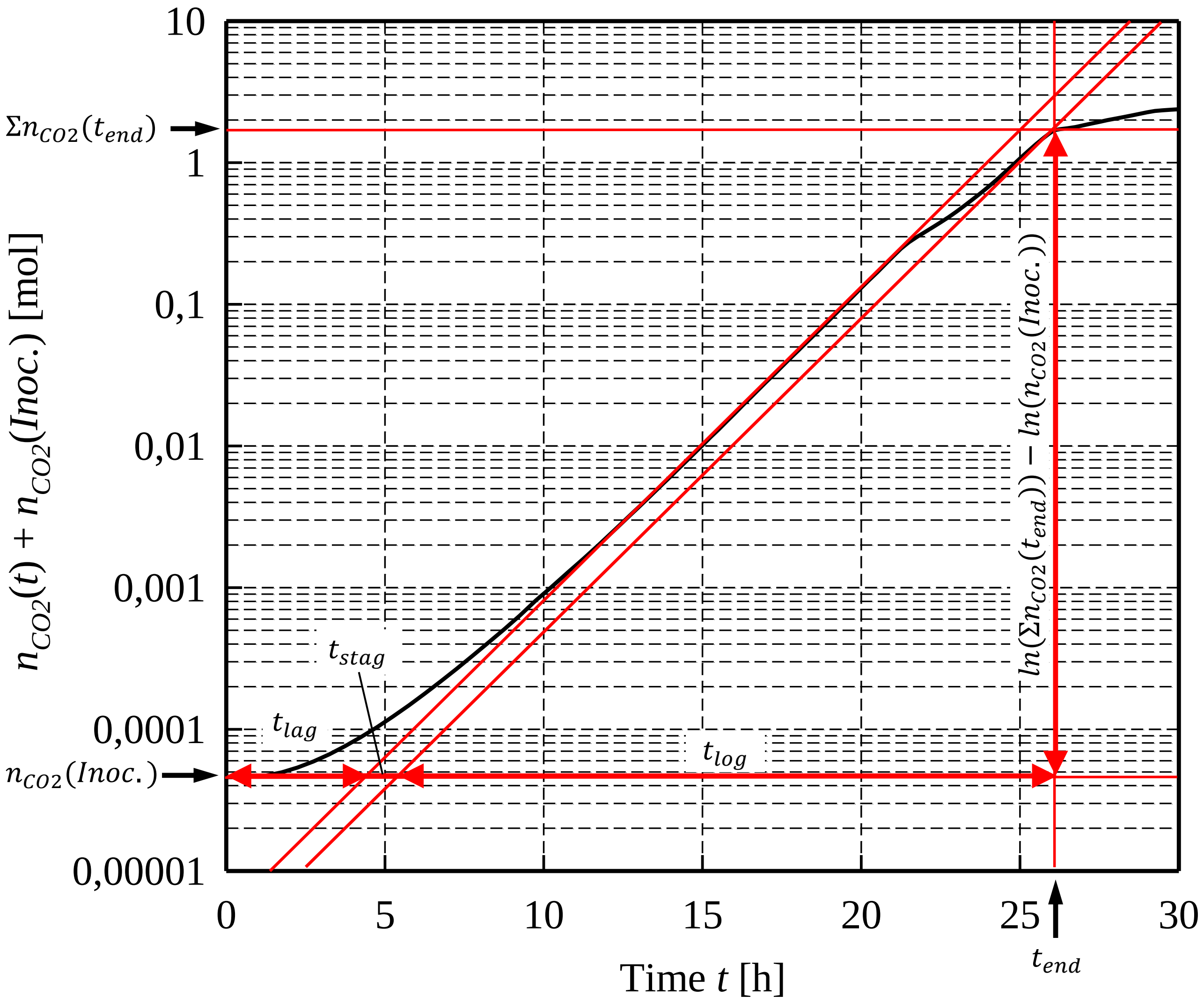

Fig. OR3.1 Amount of cumulatively formed CO_2 including the CO_2 released at formation of the inoculum depending on the cultivation time during the aerobic batch cultivation of *K. marxianus* DSM 5422 in a stirred bioreactor using 1 L DWP-based medium with a DWP content of 0.75 L L^{-1}, supplemented with urea and trace-element solution with iron; Cultivation at 40 °C, an aeration rate of 180 L h^{-1} at standard conditions and pH 5.1

After the inoculation of the cultivation medium at $t = 0$, the quantity of the CO_2, $\Sigma n_{CO2}(t)$, amounted to the CO_2 which was released during formation of the inoculum (here, $n_{CO2}(Inoc.) = 4.6 \cdot 10^{-5}$ mol CO_2). In der following lag period, the CO_2 formation was at first low but then intensified with time. Later, the

$\Sigma n_{CO2}(t)$ curve approached a straight line which marks the exponential growth phase. After consumption of 15% of the utilizable carbon sources (at $\Sigma n_{CO2}(t) \approx 0.25$ mol), the lactate became depleted and resulted in a temporary stagnation of the CO_2 release for the duration $t_{Stag.}$. The rate of CO_2 formation approached once more the exponential mode till depletion of the sugar at t_{end}, where the $\Sigma n_{CO2}(t)$ curve bended.

According to the diagram, the process time till sugar depletion is the sum of t_{lag}, t_{log} and t_{stag}:

$$t_{end} = t_{lag} + t_{log} + t_{stag} \quad \text{(OR3.7)}$$

And for the period of the exponential growth, the following equation becomes valid:

$$\mu = \frac{ln(\Sigma n_{CO2}(t_{end})) - ln(n_{CO2}(Inoc.))}{t_{log}} \quad \text{(OR3.8)}$$

Substitution of t_{log} by Eq. (OR3.7) results in:

$$\mu = \frac{ln(\Sigma n_{CO2}(t_{end})) - ln(n_{CO2}(Inoc.))}{t_{end} - t_{lag} - t_{Stag.}} \quad \text{(OR3.9)}$$

In this way, the parameters t_{lag} and μ can be easily determined from measured $\Sigma n_{CO2}(t)$ courses. Alternatively, these parameters could also be derived from measured $C_X(t)$ data, but biomass data are more prone to error, especially at the beginning of the cultivation process when the $C_X(t)$ values are very small. This means that parameters derived from the measured CO_2 content of the exhaust gas are much more reliable.

Table OR3.1 Parameters of four growth experiments performed with differently diluted DWP as stated in the figure capture of Figure OR3.1; amount of CO_2 connected with the inoculum uniformly $n_{CO2}(Inoc.) = 4.6 \cdot 10^{-5}$ mol

DWP in medium [L L^{-1}]	Mineral content [g L^{-1}]	Process duration t_{end} [h]	Stagnation period t_{stag} [h]	lag period t_{lag} [h]	formed CO_2 $\Sigma n_{CO2}(t_{end})$ [mol]	Specific growth rate μ [h^{-1}]
0.10	5.8	13.92	0.6	0.00	0.25	0.646
0.50	26.4	18.93	1.2	0.61	1.22	0.595
0.75	39.0	26.06	1.0	4.32	1.80	0.510
1.00	51.6	57.60	1.0	28.02	2.07	0.375

The finally formed CO_2 well correlates with the content of utilizable carbon sources of the culture media, with exception of the non-diluted medium where significant amounts of ethanol were formed (flow of carbon not only into the biomass and CO_2 but also into synthesized product).

Results and Discussion

The more minerals the DWP-based media contained, the lower the growth rate of *K. marxianus* DSM 5422 was (Table OR3.1). The effect of minerals on the growth rate exhibited a typical progressive inhibition kinetics. Several inhibition models have been reviewed by Urit et al. (2013) where the model of Luong (1985) described the experimental findings best:

$$\mu = \mu(C_I{=}0) \cdot (1 - (C_I/C_{I,max})^n) \quad \text{(OR3.10)}$$

Herein, $\mu(C_I{=}0)$ denotes the growth rate without inhibition, C_I is the inhibitor concentration, $C_{I,max}$ denotes the inhibitor concentration at which the growth rate just becomes zero, and parameter n determines the curvature of the $\mu(C_I)$ function. The best data fit was obtained for $\mu(C_I{=}0) = 0.65$ h^{-1}, $C_{I,max} = 73.8$ g L^{-1}, and $n = 2.4$ (Fig. 1a).

The increasing mineral content also unfavorably prolonged the growth period (Fig. 1b). The data processing showed that this prolongation was not only caused by the reduced growth rate alone but was also reasoned by an extended lag phase. Here, the term 'lag phase' is not used *sensu stricto* (i.e., as the period for synthesis of new enzymes via transcription and translation) but in a broader sense, meaning a general delay in growth without specifying the reason. In medium with 50% DWP, the lag phase lasted 0.6 h and was negligible, in medium with 75% DWP, the lag phase was 4.3 h and thus still reasonable, but in pure DWP, the lag phase lasted 28 h being unacceptable for practical applications (Fig. 1b). The transition from a low salt (YPC plate culture) to a high salt environment (DWP media) certainly caused a hyperosmotic shock. Such osmostress results in rapid cell shrinking slowing down intracellular diffusion and cell growth (Babazadeh et al. 2017). The period to adapt to the new environmental conditions depends on the change of salt concentration (Babazadeh et al. 2013). Such delay in growth has also been observed for *K. marxianus* DSM 5422 at inhibition by acetate (Martynova et al. 2016). The resilience of yeasts against DWP-borne minerals could be increased by artificial laboratory evolution.

Nomenclature

Symbol	Unit	Description
$C_X(t)$	g L^{-1}	Biomass concentration
$n_{CO2}(Inoc.)$	mol	Amount of CO_2 microbially produced during formation of the inoculum
$n_{CO2}(t)$	mol	Amount of microbially formed CO_2 till a given time
$\dot{n}_{CO2}(t)$	mol h^{-1}	Molar flow of microbially formed CO_2 during the cultivation
t	h	Process time
t_{end}	h	Moment of the depletion of sugar in the cultivation medium
t_{lag}	h	lag period of the cultivation process
t_{log}	h	Period of exponential growth phase
t_{stag}	h	Period of the temporary stagnation phase
V_L	L	Volume of the cultivation medium
$Y_{CO2/X}$	mol g^{-1}	Moles of microbially formed CO_2 per gram of formed biomass
μ	h^{-1}	Specific growth rate
$\Sigma n_{CO2}(t)$	mol	$= n_{CO2}(Inoc.) + n_{CO2}(t)$

References

Babazadeh R, Adiels CB, Smedh M, Petelenz-Kurdziel E, Goksör M, Hohmann S (2013). Osmostress-induced cell volume loss delays yeast Hog1 signaling by limiting diffusion processes and by Hog1-specific effects. PLoS One 8(11):e80901.

Babazadeh R, Lahtvee P-J, Adiels CB, Goksör M, Nielsen JB, Hohmann S (2017). The yeast osmostress response is carbon source dependent. Sci Rep 7(1):990.

Löser C, Kupsch C, Walther T, Hoffmann A (2021) A new approach for balancing the microbial synthesis of ethyl acetate and other volatile metabolites during aerobic bioreactor cultivations. Eng Life Sci 21:137–153.

Luong JHT (1985) Kinetics of ethanol inhibition in alcohol fermentation. Biotechnol Bioeng 27:280–285.

Martynova J, Kokina A, Kibilds J, Liepins J, Scerbaka R, Vigants A (2016) Effects of acetate on *Kluyveromyces marxianus* DSM 5422 growth and metabolism. Appl Microbiol Biotechnol 100:4585–4594.

Urit T, Manthey R, Bley T, Löser C (2013) Formation of ethyl acetate by *Kluyveromyces marxianus* on whey: Influence of aeration and inhibition of yeast growth by ethyl acetate. Eng Life Sci 13:247–260.
