# Supplementary material for: Utilization of delactosed whey permeate for the synthesis of ethyl acetate with Kluyveromyces marxianus
Source: Appl Microbiol Biotechnol. 2023 Feb 14;107(5-6):1635–48. doi: 10.1007/s00253-023-12419-1 (PMC10006051; doi:10.1007/s00253-023-12419-1)
Supplement: Supplementary file 4 — Supplementary file4 (PDF 621 KB) [file 253_2023_12419_MOESM4_ESM.pdf]

Online Resource 4

Batch cultivation of *K. marxianus* DSM 5422 in DWP^{-Fe} medium at pH 5.9

Title: Utilization of delactosed whey permeate for the synthesis of ethyl acetate with *Kluyveromyces marxianus*

Journal: Applied Microbiology and Biotechnology

Authors: Andreas Hoffmann [1], Alexander Franz [1,2], Thomas Walther [1], Christian Löser [1]

[1] Chair of Bioprocess Engineering, Institute of Natural Materials Technology, Technische Universität Dresden, 01062 Dresden, Germany

[2] Chair of Biophysical Chemistry, Institute of Biochemistry, University of Leipzig, 04103 Leipzig, Germany

Corresponding author: Dr. habil. Christian Löser (christian-loeser@tu-dresden.de)

Table OR4.1 Parameters of cell growth and product synthesis during the aerobic batch cultivation of *K. marxianus* DSM 5422 in a stirred bioreactor under iron-limited conditions using DWP^{-Fe} medium; Cultivation at 40 °C, aeration with 60 L h^{-1}, and pH 5.9; Further details of the cultivations are given in the caption of Fig. OR4.1

Process parameter		Value
Process time till depletion of sugars	[h]	48.8
Average respiratory quotient, RQ	[mol mol^{-1}]	1.38
Final biomass concentration	[g L^{-1}]	13.60
Maximum $C_{EA,G}$	[mg L^{-1}]	34.9
Maximum $C_{EA,L}$	[g L^{-1}]	1.67
Mass of formed ethyl acetate, m_{EA}	[g]	13.9
Mass of stripped ethyl acetate	[g]	13.7
Maximum R_{EA}	[g $L^{-1}h^{-1}$]	2.72
Maximum r_{EA}	[g $g^{-1}h^{-1}$]	0.33
Overall yield of ethyl acetate, $Y_{EA/S}$	[g g^{-1}]	0.185
$Y_{EA/S}$-$Y_{EA/S,max}$ ratio	[%]	36.0
Selectivity of ester formation	[g g^{-1}]	0.604
Selectivity of ester stripping	[g g^{-1}]	0.722
Maximum $C_{EtOH,G}$	[mg L^{-1}]	2.20
Maximum $C_{EtOH,L}$	[g L^{-1}]	4.96
Mass of formed ethanol, m_{EtOH}	[g]	5.66
Mass of stripped ethanol	[g]	1.89
Maximum R_{EtOH}	[g $L^{-1}h^{-1}$]	0.66
Maximum r_{EtOH}	[g $g^{-1}h^{-1}$]	1.06
Overall yield of ethanol, $Y_{EtOH/S}$	[g g^{-1}]	0.08
Maximum $C_{AA,G}$	[mg L^{-1}]	3.08
Maximum $C_{AA,L}$	[g L^{-1}]	0.42
Mass of formed acetaldehyde, m_{AA}	[g]	2.16
Mass of stripped acetaldehyde	[g]	2.10
Maximum R_{AA}	[g $L^{-1}h^{-1}$]	0.38
Maximum r_{AA}	[g $g^{-1}h^{-1}$]	0.15
Overall yield of acetaldehyde, $Y_{AA/S}$	[g g^{-1}]	0.04
Maximum $C_{Ac,L}$	[g L^{-1}]	1.41
Mass of formed acetate, m_{Ac}	[g]	1.28
Overall yield of acetate, $Y_{Ac/S}$	[g g^{-1}]	0.02

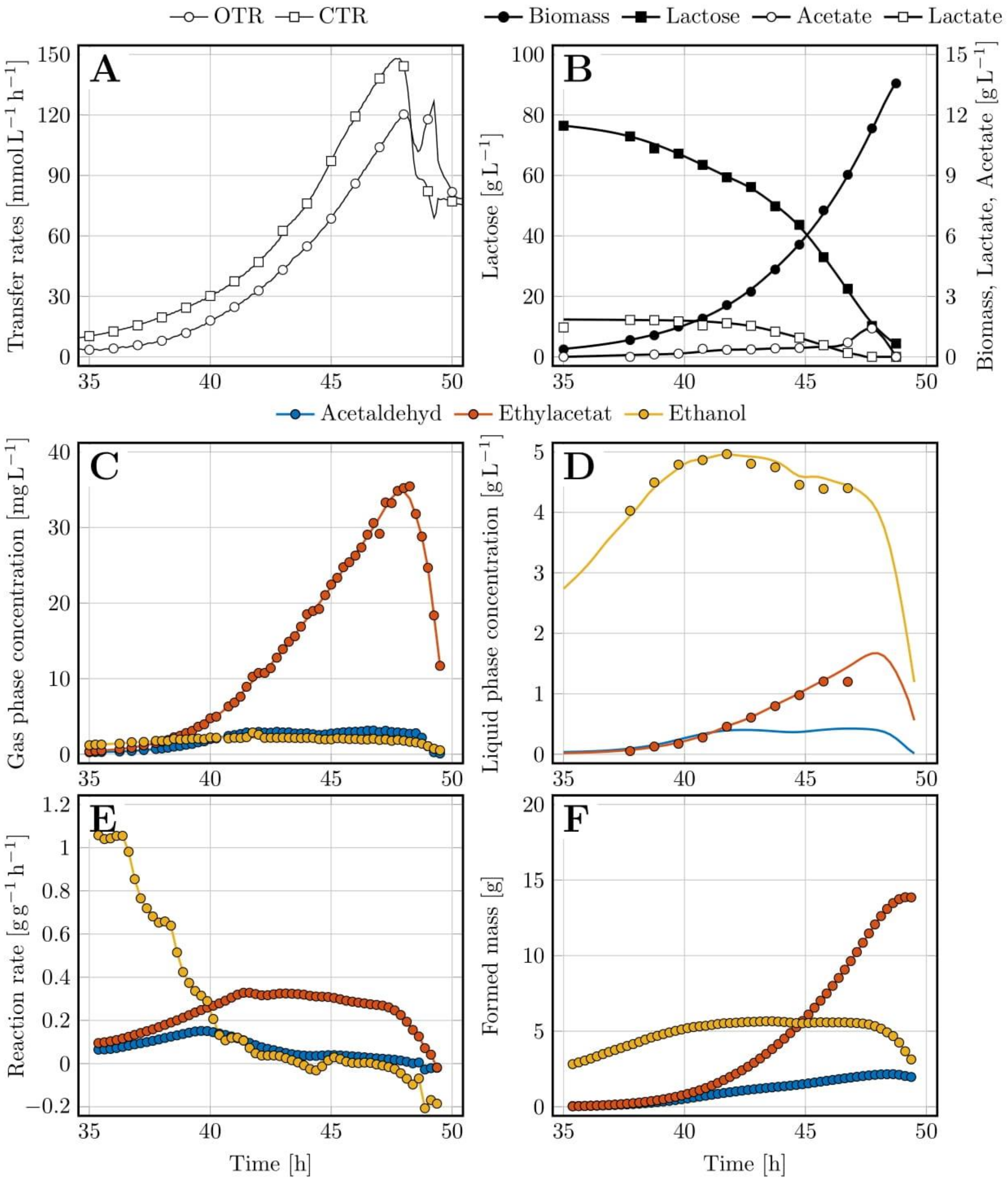

Fig. OR4.1 (A) Oxygen transfer rate (OTR) and CO_2 transfer rate (CTR); (B) Lactose, biomass, acetate and lactate concentration; (C) Gas phase concentrations, (D) Liquid phase concentrations, (E) Biomass-specific reaction rates, and (F) masses of formed ethyl acetate, ethanol and acetaldehyde during the aerobic batch cultivation of *K. marxianus* DSM 5422 under iron-limited conditions in a stirred bioreactor using 1 L DWP^{-Fe} medium; Cultivation at 40 °C, aeration with 60 L h^{-1}, and pH 5.9
