# Supplementary material for: Utilization of delactosed whey permeate for the synthesis of ethyl acetate with Kluyveromyces marxianus
Source: Appl Microbiol Biotechnol. 2023 Feb 14;107(5-6):1635–48. doi: 10.1007/s00253-023-12419-1 (PMC10006051; doi:10.1007/s00253-023-12419-1)
Supplement: Supplementary file 5 — Supplementary file5 (PDF 625 KB) [file 253_2023_12419_MOESM5_ESM.pdf]

Online Resource 5

Fed-batch cultivation of *K. marxianus* DSM 5422 in DWP^{-Fe} medium at pH 5.3

Title: Utilization of delactosed whey permeate for the synthesis of ethyl acetate with *Kluyveromyces marxianus*

Journal: Applied Microbiology and Biotechnology

Authors: Andreas Hoffmann [1], Alexander Franz [1,2], Thomas Walther [1], Christian Löser [1]

[1] Chair of Bioprocess Engineering, Institute of Natural Materials Technology, Technische Universität Dresden, 01062 Dresden, Germany

[2] Chair of Biophysical Chemistry, Institute of Biochemistry, University of Leipzig, 04103 Leipzig, Germany

Corresponding author: Dr. habil. Christian Löser (christian-loeser@tu-dresden.de)

Table OR5.1 Parameters of cell growth and product synthesis during aerobic fed-batch cultivations of *K. marxianus* DSM 5422 in a stirred bioreactor under iron-limited conditions using DWP^{-Fe} medium; Cultivation at 40 °C and aeration with 60 L h^{-1}; The process started with 1 L DWP^{-Fe} medium at pH 5.1 and was continued at pH 5.3 after the CO_2 content of the exhaust gas exceeded 0.001 L L^{-1}; The process was continued by feeding another 1 L DWP^{-Fe} medium with a rate of 0.45 L h^{-1}

Process parameter		Value
Process time till depletion of sugars	[h]	24.8
Final proportion of living cells	[%]	95.5
Average respiratory quotient, RQ	[mol mol^{-1}]	1.72
Final biomass concentration	[g L^{-1}]	6.70
Maximum $C_{EA,G}$	[mg L^{-1}]	110.0
Maximum $C_{EA,L}$	[g L^{-1}]	4.67
Mass of formed ethyl acetate, m_{EA}	[g]	50.9
Mass of stripped ethyl acetate	[g]	47.4
Maximum R_{EA}	[g $L^{-1}h^{-1}$]	5.03
Maximum r_{EA}	[g $g^{-1}h^{-1}$]	0.84
Selectivity of ester formation	[g g^{-1}]	0.700
Selectivity of ester stripping	[g g^{-1}]	0.962
Maximum $C_{EtOH,G}$	[mg L^{-1}]	3.45
Maximum $C_{EtOH,L}$	[g L^{-1}]	5.84
Mass of formed ethanol, m_{EtOH}	[g]	12.21
Mass of stripped ethanol	[g]	0.93
Maximum R_{EtOH}	[g $L^{-1}h^{-1}$]	2.23
Maximum r_{EtOH}	[g $g^{-1}h^{-1}$]	0.38
Maximum $C_{AA,G}$	[mg L^{-1}]	4.99
Maximum $C_{AA,L}$	[g L^{-1}]	0.65
Mass of formed acetaldehyde, m_{AA}	[g]	1.96
Mass of stripped acetaldehyde	[g]	0.94
Maximum R_{AA}	[g $L^{-1}h^{-1}$]	0.41
Maximum r_{AA}	[g $g^{-1}h^{-1}$]	0.06
Maximum $C_{Acetate,L}$	[g L^{-1}]	4.35
Mass of formed acetate, $m_{Acetate}$	[g]	7.70

Product yields could not be calculated for this experiment because of missing analyses of the initial sugar content in the used DWP^{-Fe} medium.

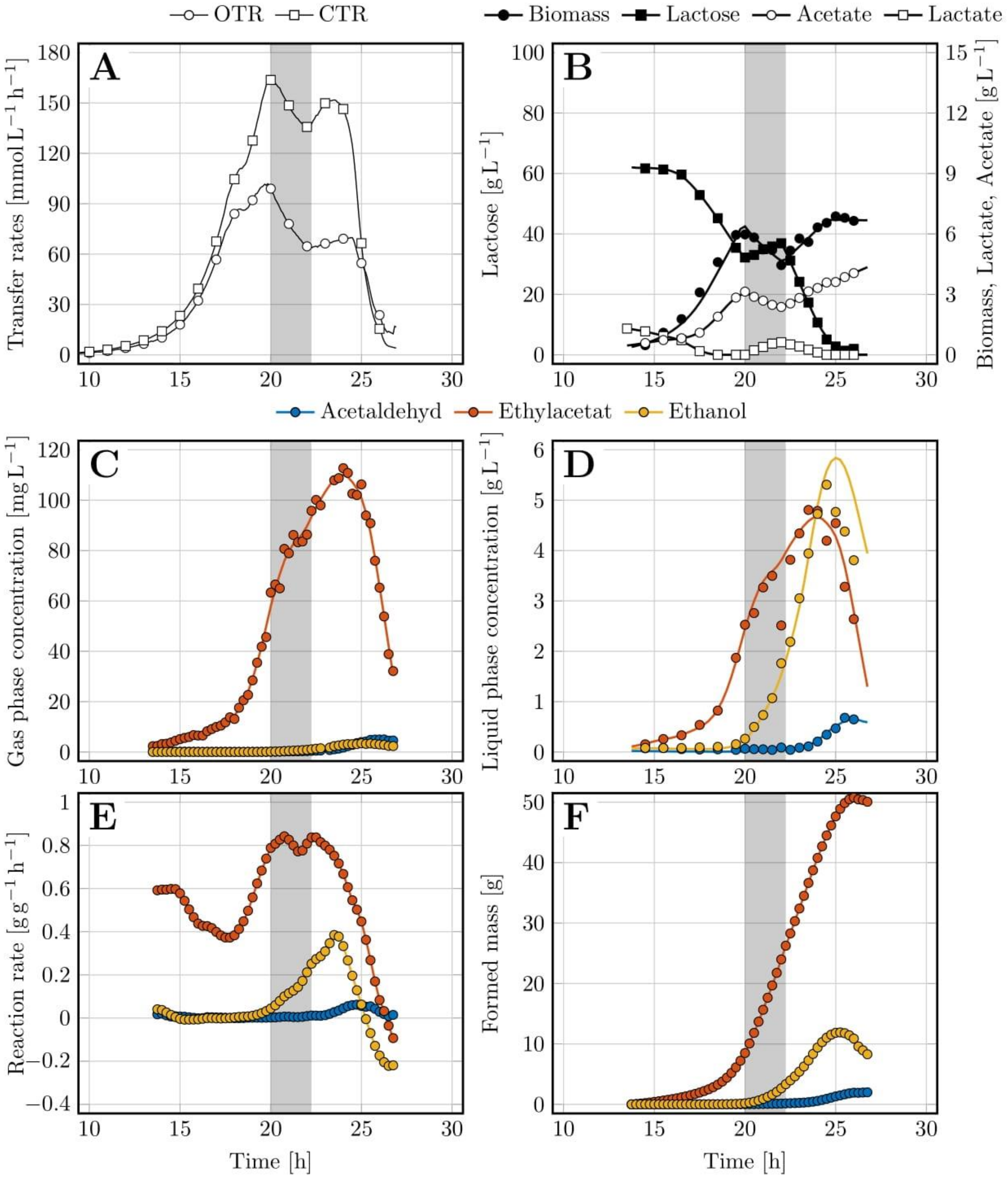

Fig. OR5.1 (A) Oxygen transfer rate (OTR) and CO_2 transfer rate (CTR); (B) Lactose, biomass, acetate and lactate concentration; (C) Gas phase concentrations, (D) Liquid phase concentrations, (E) Biomass-specific reaction rates, and (F) Masses of formed ethyl acetate, ethanol and acetaldehyde during the aerobic fed-batch cultivation of *K. marxianus* DSM 5422 under iron-limited conditions in a stirred bioreactor using DWP^{-Fe} medium; Cultivation at 40 °C and aeration with 60 L h^{-1}; The process started with 1 L DWP^{-Fe} medium at pH 5.1 and was continued at pH 5.3 after the CO_2 content of the exhaust gas exceeded 0.001 L L^{-1}; The process was continued by feeding another 1 L DWP^{-Fe} medium with a rate of 0.45 L h^{-1} (marked by the grey area)
