# Supplementary material for: Utilization of delactosed whey permeate for the synthesis of ethyl acetate with Kluyveromyces marxianus
Source: Appl Microbiol Biotechnol. 2023 Feb 14;107(5-6):1635–48. doi: 10.1007/s00253-023-12419-1 (PMC10006051; doi:10.1007/s00253-023-12419-1)
Supplement: Supplementary file 6 — Supplementary file6 (PDF 627 KB) [file 253_2023_12419_MOESM6_ESM.pdf]

Online Resource 6

Repeated-batch cultivation of *K. marxianus* DSM 5422 in DWP$^{–Fe}$ medium at pH 5.4

Title: Utilization of delactosed whey permeate for the synthesis of ethyl acetate with *Kluyveromyces marxianus*

Journal: Applied Microbiology and Biotechnology

Authors: Andreas Hoffmann [1], Alexander Franz [1,2], Thomas Walther [1], Christian Löser [1]

[1] Chair of Bioprocess Engineering, Institute of Natural Materials Technology, Technische Universität Dresden, 01062 Dresden, Germany

[2] Chair of Biophysical Chemistry, Institute of Biochemistry, University of Leipzig, 04103 Leipzig, Germany

Corresponding author: Dr. habil. Christian Löser (christian-loeser@tu-dresden.de)

Table OR6.1 Parameters of cell growth and product synthesis during aerobic fed-batch cultivations of *K. marxianus* DSM 5422 in a stirred bioreactor under iron-limited conditions using DWP$^{–Fe}$ medium; Cultivation at 40 °C and aeration with 120 L h$^{–1}$; The process started with 1 L DWP$^{–Fe}$ medium at pH 5.1 and was continued at pH 5.4 after the CO_2 content of the exhaust gas exceeded 0.001 L L$^{–1}$; After depletion of sugar at 24.4 h, 0.9 L cell suspension was removed and replaced by 0.9 L fresh DWP$^{–Fe}$ medium

Process parameter		Value
Process time till depletion of sugars	[h]	32.1
Final proportion of living cells	[%]	97.3
Average respiratory quotient, RQ	[mol mol$^{–1}$]	1.31
Final biomass concentration	[g L$^{–1}$]	10.1
Maximum $C_{EA,G}$	[mg L$^{–1}$]	108.8
Maximum $C_{EA,L}$	[g L$^{–1}$]	4.62
Mass of formed ethyl acetate, m_{EA}	[g]	45.2
Mass of stripped ethyl acetate	[g]	41.2
Maximum R_{EA}	[g L$^{–1}$h$^{–1}$]	7.97
Maximum r_{EA}	[g g$^{–1}$h$^{–1}$]	0.94
Selectivity of ester formation	[g g$^{–1}$]	0.909
Selectivity of ester stripping	[g g$^{–1}$]	0.986
Maximum $C_{EtOH,G}$	[mg L$^{–1}$]	0.92
Maximum $C_{EtOH,L}$	[g L$^{–1}$]	1.77
Mass of formed ethanol, m_{EtOH}	[g]	2.93
Mass of stripped ethanol	[g]	0.30
Maximum R_{EtOH}	[g L$^{–1}$h$^{–1}$]	0.96
Maximum r_{EtOH}	[g g$^{–1}$h$^{–1}$]	0.11
Maximum $C_{AA,G}$	[mg L$^{–1}$]	0.74
Maximum $C_{AA,L}$	[g L$^{–1}$]	0.10
Mass of formed acetaldehyde, m_{AA}	[g]	0.43
Mass of stripped acetaldehyde	[g]	0.28
Maximum R_{AA}	[g L$^{–1}$h$^{–1}$]	0.18
Maximum r_{AA}	[g g$^{–1}$h$^{–1}$]	0.02
Maximum $C_{Acetate,L}$	[g L$^{–1}$]	1.13
Mass of formed acetate, $m_{Acetate}$	[g]	1.18

Product yields could not be calculated for this experiment because of missing analyses of the sugar content in the used DWP$^{–Fe}$ medium.

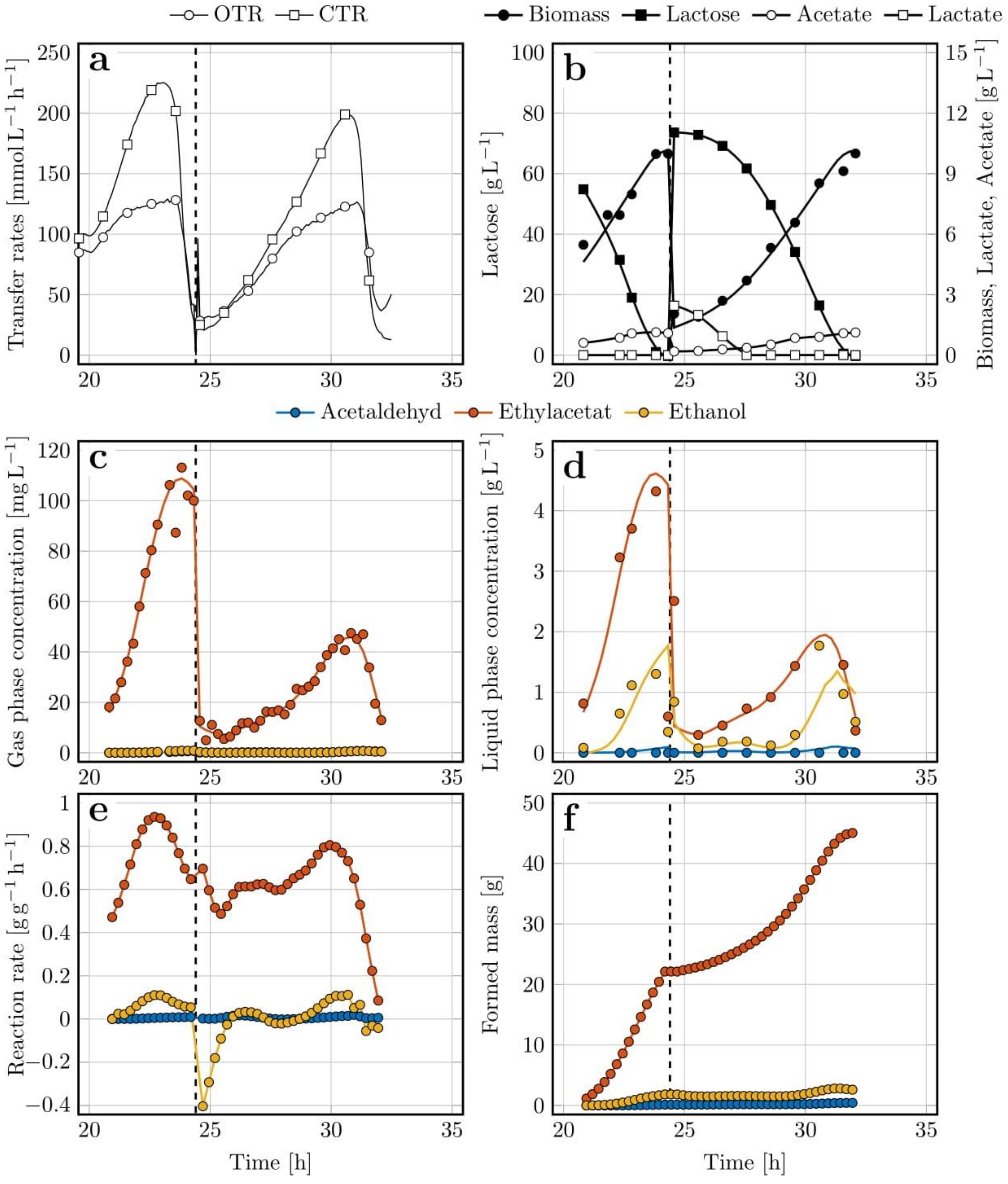

Fig. OR6.1 (A) Oxygen transfer rate (OTR) and CO_2 transfer rate (CTR); (B) Lactose, biomass, acetate and lactate concentration; (C) Gas phase concentrations, (D) Liquid phase concentrations, (E) Biomass-specific reaction rates, and (F) Masses of formed ethyl acetate, ethanol and acetaldehyde during the aerobic repeated-batch cultivation of *K. marxianus* DSM 5422 under iron-limited conditions in a stirred bioreactor using DWP^{-Fe} medium; Cultivation at 40 °C and aeration with 60 and 120 L h^{-1} for the first and second batch, respectively; The process started with 1 L DWP^{-Fe} medium at pH 5.1 and was continued at pH 5.4 after the CO_2 content of the exhaust gas exceeded 0.001 L L^{-1}; After depletion of sugar at 21.4 h, 0.9 L cell suspension was removed and replaced by 0.9 L fresh DWP^{-Fe} medium (marked by a dashed line)
