# Supplementary material for: Utilization of delactosed whey permeate for the synthesis of ethyl acetate with Kluyveromyces marxianus
Source: Appl Microbiol Biotechnol. 2023 Feb 14;107(5-6):1635–48. doi: 10.1007/s00253-023-12419-1 (PMC10006051; doi:10.1007/s00253-023-12419-1)
Supplement: Supplementary file 7 — Supplementary file7 (PDF 388 KB) [file 253_2023_12419_MOESM7_ESM.pdf]

Online Resource 7

Comparison between iron-limited and non-limited cultivations of *K. marxianus* DSM 5422 in DWP medium

Title: Utilization of delactosed whey permeate for the synthesis of ethyl acetate with *Kluyveromyces marxianus*

Journal: Applied Microbiology and Biotechnology

Authors: Andreas Hoffmann [1], Alexander Franz [1,2], Thomas Walther [1], Christian Löser [1]

[1] Chair of Bioprocess Engineering, Institute of Natural Materials Technology, Technische Universität Dresden, 01062 Dresden, Germany

[2] Chair of Biophysical Chemistry, Institute of Biochemistry, University of Leipzig, 04103 Leipzig, Germany

Corresponding author: Dr. habil. Christian Löser (christian-loeser@tu-dresden.de)

Here, we compare the aerobic growth of the yeast *K. marxianus* DSM 5422 in 1 L whey-based DWP medium with or without a supplementation of iron. These cultivations were performed in a stirred bioreactor at 40 °C and pH values of 5.1 (Fig. OR7.1) or 5.9 (Fig. OR7.2). The aeration of the bioreactor occurred with 60 L h^{-1} in iron-limited processes or with 180 L h^{-1} in non-limited processes to cover the higher demand for oxygen (both aeration rates are given for 0 °C and 101325 Pa).
DWP medium with supplementation of iron (DWP^{+Fe} medium) enabled a quick and nearly exponential growth, but the formation of ethyl acetate was marginal. The biomass growth in DWP medium without a supplementation of iron (DWP^{-Fe} medium), in contrast, was diminished with a switch from an initially exponential to a linear growth characteristic, and high amounts of ester were formed. Moreover, a significant lag phase occurred under iron limitation but only when the pH value was 5.9 (compare Figs. OR7.2a with Fig. OR7.1a).

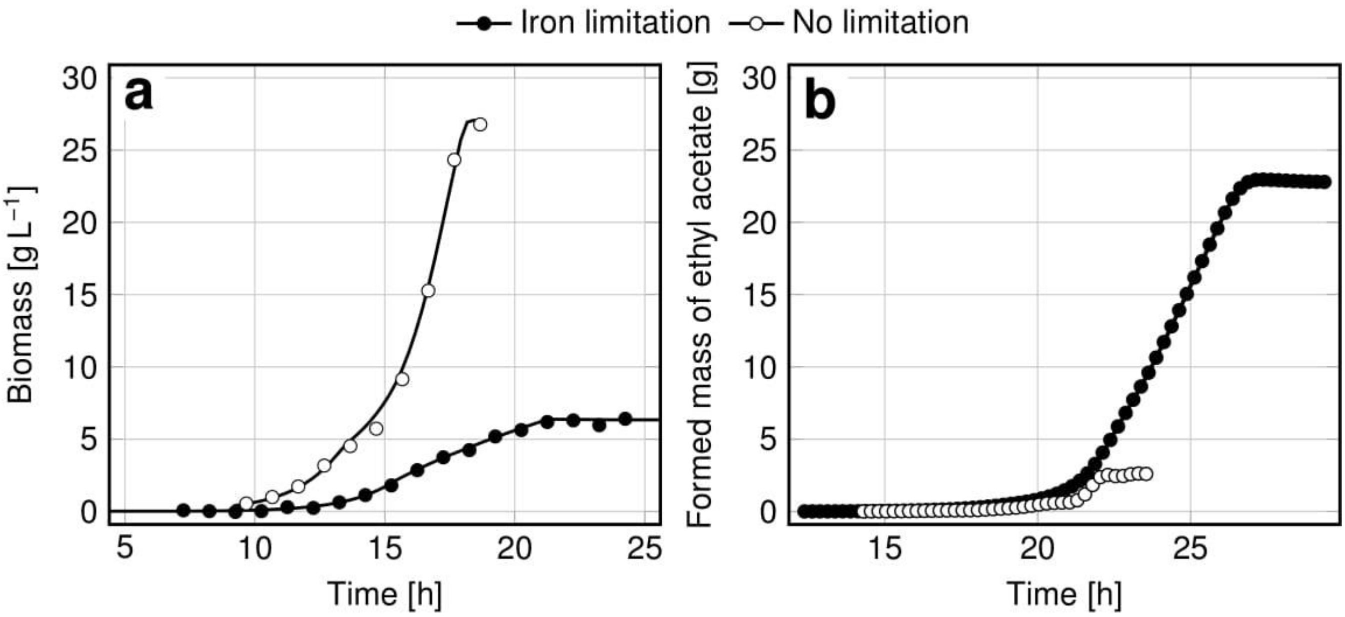

Fig. OR7.1 Comparison between iron-limited and non-limited cultivation of *K. marxianus* DSM 5422 in DWP medium. (a) Biomass concentration; (b) Mass of formed ethyl acetate. The cultivations of *K. marxianus* DSM 5422 were conducted as batch processes with 1 L DWP medium with (no limitation) and without supplementation iron (iron limitation). The cultivations were carried out in a stirred and aerated bioreactor at 40 °C, pH 5.1 and aeration rate of 60 and 180 L h^{-1} for the iron-limited and non-limited process, respectively

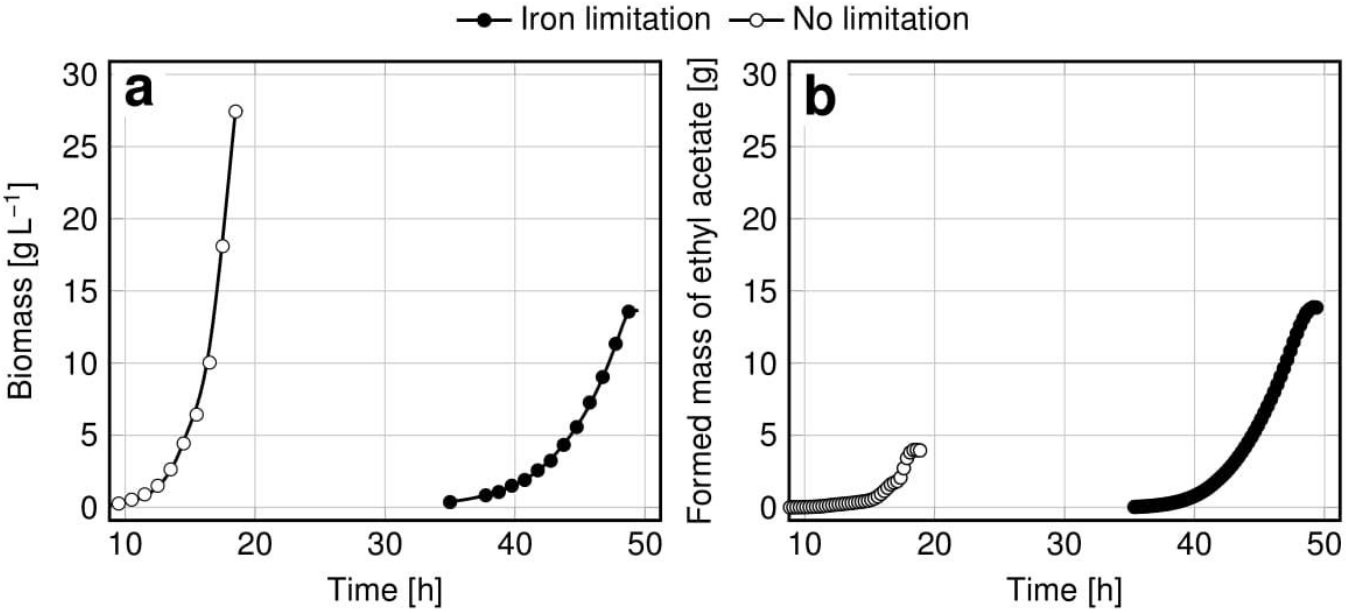

Fig. OR7.2 Comparison between iron-limited and non-limited cultivation of *K. marxianus* DSM 5422 in DWP medium. (a) Biomass concentration; (b) Mass of formed ethyl acetate. The cultivations of *K. marxianus* DSM 5422 were conducted as batch processes with 1 L DWP medium with (no limitation) and without supplementation iron (iron limitation). The cultivations were carried out in a stirred and aerated bioreactor at 40 °C, pH 5.9 and aeration rate of 60 and 180 L h^{-1} for the iron-limited and non-limited process, respectively

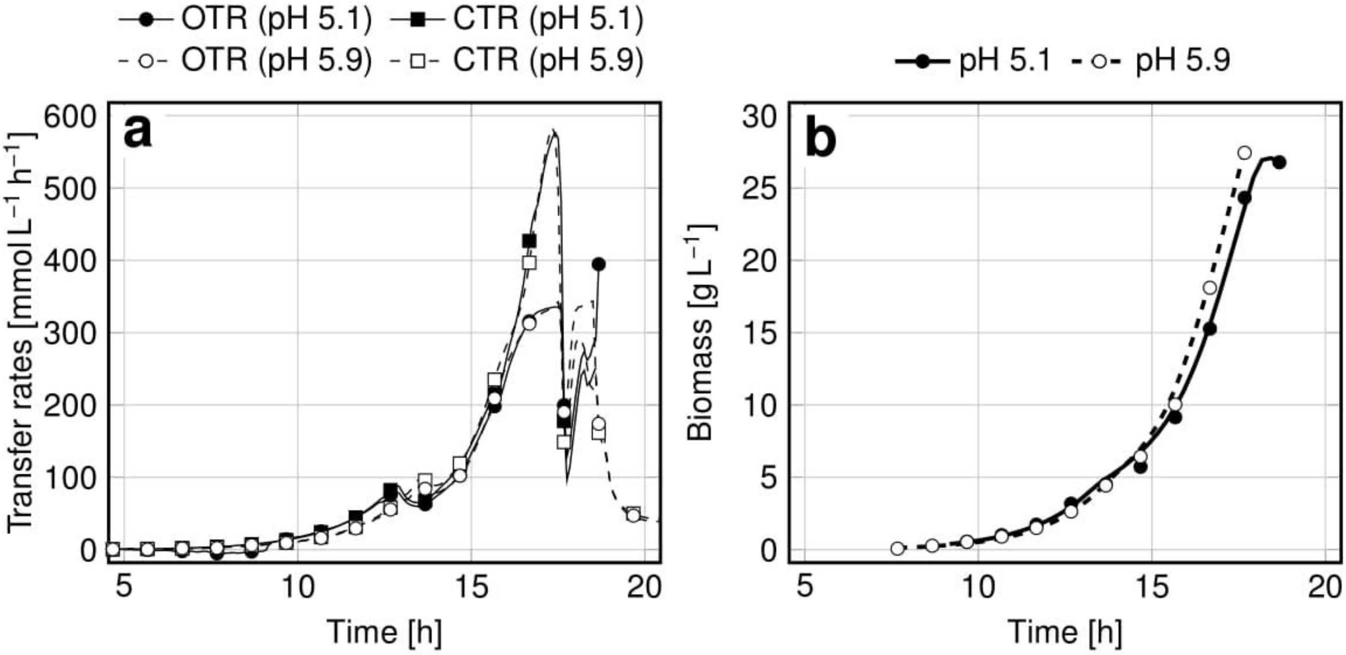

Fig. OR7.3 Comparison between non-limited cultivations of *K. marxianus* DSM 5422 in DWP medium. (a) CTR and OTR; (b) Biomass concentration. The cultivations of *K. marxianus* DSM 5422 were conducted as batch processes with 1 L DWP medium with supplementation of iron (no limitation). The cultivations were carried out in a stirred and aerated bioreactor at 40 °C, pH 5.1 or pH 5.9 180 L h^{-1}.
